# Enter the Dragon: The Dynamic and Multifunctional Evolution of Anguimorpha Lizard Venoms

**DOI:** 10.3390/toxins9080242

**Published:** 2017-08-06

**Authors:** Ivan Koludarov, Timothy NW Jackson, Bianca op den Brouw, James Dobson, Daniel Dashevsky, Kevin Arbuckle, Christofer J. Clemente, Edward J. Stockdale, Chip Cochran, Jordan Debono, Carson Stephens, Nadya Panagides, Bin Li, Mary-Louise Roy Manchadi, Aude Violette, Rudy Fourmy, Iwan Hendrikx, Amanda Nouwens, Judith Clements, Paolo Martelli, Hang Fai Kwok, Bryan G. Fry

**Affiliations:** 1 Venom Evolution Lab, School of Biological Sciences, University of Queensland, St. Lucia, QLD 4072, Australia; jcoludar@gmail.com (I.K.); tnwjackson@gmail.com (T.N.W.J.); b.opdenbrouw@uq.net.au (B.o.d.B.); james.dobson@uqconnect.edu.au (J.D.); danieldashevsky@gmail.com (D.D.); jordan.debono@uqconnect.edu.au (J.D.); nadya.panagides@gmail.com (N.P.); iwanhx@yahoo.com (I.H.); 2 Australian Venom Research Unit, School of Biomedical Sciences, Level 2 Medical Building, University of Melbourne, Melbourne, VIC 3010, Australia; 3 Department of Biosciences, College of Science, Swansea University, Swansea SA2 8PP, UK; kevin.arbuckle@swansea.ac.uk; 4 University of the Sunshine Coast, School of Science and Engineering, Sippy Downs, QLD 4558, Australia; cclement@usc.edu.au; 5 Gradient Scientific and Technical Diving, Rye, VIC 3941, Australia; evolution_bug@hotmail.com; 6 Department of Earth and Biological Sciences, Loma Linda University, Loma Linda, CA 92350, USA; skipc8384@hotmail.com; 7 School of Biomedical Sciences, Queensland University of Technology, Brisbane, QLD 4001, Australia; cr.stephens@qut.edu.au (C.S.); j.clements@qut.edu.au (J.C.); 8 Faculty of Health Sciences, University of Macau, Avenida da Universidade, Taipa, Macau; yb47627@umac.mo (B.L.); hfkwok@umac.mo (H.F.K.); 9 School of Biomedical Sciences, University of Queenslnd, St. Lucia, QLD 4072, Australia; m.roymanchadi@uq.edu.au; 10 Alphabiotoxine Laboratory sprl, Barberie 15, 7911 Montroeul-au-bois, Belgium; rd.science@alphabiotoxine.be (A.V.); info@alphabiotoxine.be (R.F.); 11 School of Chemistry and Molecular Biology, University of Queenslnd, St. Lucia, QLD 4072, Australia; a.nouwens@uq.edu.au; 12 Veterinary Department, Ocean Park, Aberdeen, Hong Kong; paolo.martelli@oceanpark.com.hk

**Keywords:** venom, lizard, evolution, Toxicofera, coagulation, fibrinogen, proteomics

## Abstract

While snake venoms have been the subject of intense study, comparatively little work has been done on lizard venoms. In this study, we have examined the structural and functional diversification of anguimorph lizard venoms and associated toxins, and related these results to dentition and predatory ecology. Venom composition was shown to be highly variable across the 20 species of *Heloderma*, *Lanthanotus*, and *Varanus* included in our study. While kallikrein enzymes were ubiquitous, they were also a particularly multifunctional toxin type, with differential activities on enzyme substrates and also ability to degrade alpha or beta chains of fibrinogen that reflects structural variability. Examination of other toxin types also revealed similar variability in their presence and activity levels. The high level of venom chemistry variation in varanid lizards compared to that of helodermatid lizards suggests that venom may be subject to different selection pressures in these two families. These results not only contribute to our understanding of venom evolution but also reveal anguimorph lizard venoms to be rich sources of novel bioactive molecules with potential as drug design and development lead compounds.

## 1. Introduction

Reptile venom evolution in general is an area of controversy and lizard venom is particularly contentious. Anecdotal data on complications following *V. komodoensis* bites triggered scientific interest and were at first explained by the potential existence of pathogenic bacteria unique to the lizards’ oral flora. The origin of this idea dates back to folk myths; however, Auffenberg is often quoted as the originator of it. In his monumental 1981 study [1], he reports the presence of *Staphylococcus* sp., *Providencia* sp., *Proteus morgani* and *Proteus mirabilis* in mucoid samples from the external gum surface of the upper jaw of two freshly captured “oras” (the local name for *V. komodoensis*). In the same study, the specimen from the San Diego Zoo possessed none of these bacteria, and Auffenberg suggested that oras may depend on frequent reinfestation from carrion to replenish their “weaponised bacteria”. Though Auffenberg concludes that *Proteus*-dominated infection could be responsible for the consequences of some of the recorded bites and could potentially play an adaptive role in *V. komodoensis* ecology, he himself concludes “that the infectious feature of an ora bite is a folk myth” [1]. A more recent study [2] echoed these findings. 

It was not until very recently, however, that the idea was definitively discarded, since *V. komodoensis* oral flora turned out to be not at all dissimilar from that of any other carnivorous animal [3]. The sensational reports of buffalo dying from infection are not only exaggerations about the frequency of what is a rare event, but they are also a fundamental misreading of scenario with recent anthropogenic origins—*V. komodoensis* evolved in Australia, alongside two larger species of varanid lizard, and subsequently radiated into Indonesia [4]. Water buffalo, on the other hand, were introduced to the islands only 300 years ago by Dutch settlers. In their native environment water buffalo frequent vast marshes, whilst on the islands of Komodo and Rinca, which they share with *V. komodoensis*, the only available water sources are stagnant rocky pools measuring only 5–10 m across. *V. komodoensis* attacks upon water buffalo are invariably unsuccessful. However, subsequent to seeking refuge in watering holes filled with their own sewage, water buffalo may become infected with pathogenic bacteria that ultimately cause fatal sepsis. This scenario, in which ora attacks and buffalo deaths are temporally, but not causally, connected, compounded by the observation of oras scavenging on the carcasses of dead buffalo, likely lead to the “folk toxicology” explanation of *V. komodoensis* using bacteria as a weapon [3,5].

Further controversy concerning anguimorph lizards and the Toxicofera clade has resulted from discrepancies between phylogenies generated using morphological data alone and those generated using molecular evidence or a combination of molecular and morphological evidence [6,7,8,9]. Some adherents of morphological taxonomy have not changed their position [10] despite the growing pile of robust genetic evidence and integrated morphological/molecular analyses [11,12,13,14,15,16,17,18,19,20,21,22], in which a well-supported clade is consistently recovered containing Anguimorpha, Iguania and Serpentes [17,18,23]. This clade was given the name “Toxicofera” to reflect the presence of venom within the group. Toxinological studies have further corroborated close evolutionary relationships between snakes and anguimorph lizards, showing that Anguimorpha venom glands express proteins homologous to toxins found in the venom of front-fanged snakes [23,24,25,26,27,28,29,30,31,32,33,34]. 

Despite multiple independent studies strengthening the evidence for the phylogenetic relationship between snakes and anguimorph lizards (along with Iguania) [11,12,13,14,15,16,17,18,19,20,21,22], and the existence of unique protein-secreting oral glands as the synapomorphy of the Toxicofera clade (suggesting that the oral glands of the Toxicofera MRCA may have been “exapted” for the subsequent evolution of venom systems within certain lineages), some herpetologists continue to prefer the older, morphology-based taxonomy and reject recent developments in the evolutionary toxinology of reptiles [10,35]. Some recent publications disputing aspects of lizard venom evolution [10,35] relied upon an article with poorly resolved trees [36] that were the result of attempts to combine morphology, fossils, and genetics into a single tree rather than mapping morphology and fossils over the well-resolved genetic trees. The resulting combined trees [36] have topologies starkly different from those reconstructed using only genetic evidence [11,12,13,14,15,16,17,18,19,20,21,22], with anguimorph lizards not recovered as a monophyletic group, biasing attempts to reconstruct evolutionary histories that rely on them [10,35]. 

Further, Sweet’s assertion that the genetic support for Toxicofera as a clade is erroneous due to short-branch/long-branch attractions [10], is a flawed argument as long-branch attraction is not a serious issue for recent likelihood-based methods (as it was with older parsimony based methods), particularly when good model-selection procedures are used. Also, for DNA sequence data, long branch attraction is not expected to be an issue where more than 20% of the characters favour that topology (as is the case in the Toxicofera situation [11,12,13,14,15,16,17,18,19,20,21,22]). Indeed Sweet himself notes [10] that only a minority of the evidence cited in a review by Sites, Reeder & Wiens [37] did not support the grouping of anguimorphan and iguanian lizards as part of the Toxicofera clade. Thus Sweet’s main argument against Toxicofera is one of inertia due to the weight of history, as demonstrated by his statement “that dozens to hundreds of morphological, behavioral and ecological synapomorphies must be reversed is strong evidence of a false signal in the genes supporting the short Toxicoferan branch” [10]. Naturally the historical morphological, behavioural and ecological observations were viewed through the filter of the understanding of the organismal relationships at the time. The fact that these observations will have to be reinterpreted due to genetic evidence overturning morphology-driven taxonomy, should not be an impediment to the acceptance of newer, more robust data regarding the taxonomical arrangements. Just as the historical ecology and predatory observations of varanid lizards were viewed through the filter that these lizards were assumed to be non-venomous due to the perceived lack of venom glands, need to be reinterpreted (and even redone) in light of the discovery that varanid lizards in fact posses complex oral glands homologous to that of helodermatid lizards and secrete many of the same proteins with similar bioactivity [23,24,25,26,27,28,29,30,31,32,33,34]. Therefore, the weight of history should not stand in the way of scientific advancement. This lack of acceptance of more lizards being venomous than previously understood, is mirrored by the reluctance of some [35,38] to accept that all advanced snakes share a common venomous ancestor [25,29,34,36,39]. 

Importantly, more recent phylogenetic analyses that have combined morphological and molecular data, and simultaneously attempted to resolve the topological conflict by careful analysis and treatment of its source in the data, have again recovered Toxicofera as a robustly supported clade and uncovered new morphological synapomorphies of the clade [14,21]. Controversy regarding the phylogenetic validity of Toxicofera is often intertwined with that surrounding hypotheses concerning the evolution of venom within the clade, a situation compounded by use of the poorly defined term “Toxicofera hypothesis”. Although Hargreaves et al. (HEA [40]) used this term to refer to the “single early evolution of venom in reptiles”, the original publication concerning this “early origin” hypothesis almost exclusively prefers the term “venom system” over “venom”[23]—thus the original “Toxicofera hypothesis (of venom evolution) concerns the single early origin of the venom system, not “venom” *per se*. In that all lineages possessed uniquely derived mandibular and maxillary glands distinguished by having segregated protein and mucus secreting regions, with the enlargement of the protein-secreting region relative to that of the mucus-secreting region. More broadly, however, the term “Toxicofera hypothesis” could refer either to a hypothesis concerning the phylogenetic existence and constituency of the Toxicofera clade, or one of several interpretations of the evolutionary history of venom systems within the clade. Regardless of evidence or views on venom evolution within the clade, the weight of evidence (whether morphological or molecular) strongly supports the existence of the clade Toxicofera. It should be noted that the Hargreaves study [40] challenging the evolution of venom in lizards relied upon unreliable data such as tissues expression values up to 5000-fold in variance within a single set of replicates through to averaging only *n* = 2 including some in which one of the values was 0 (Supplementary Tables S5–S9 of [40]). Further to this the trees presented in the paper [40] Figures 5 and 6, and Supplementary Figures S1, S2, S4a, S7, S9, S12, S14 and S16–S18 contained nodes of less than 50% support or have the inclusion of nodes without support values. Had node support been presented in the main paper with the standard protocol of collapsing nodes below 50%, their trees would be largely polytomies with very little topological information. Given that many of their conclusions were based on the topology of those trees, we must consider that those conclusions were not supported by their data or analyses. Therefore their assertion that many reptile venom proteins are simply expression of ‘housekeeping genes’, and therefore calling into question the single early origin of the venom system, is not phylogenetically supported. 

Some of the controversy surrounding the “Toxicofera hypothesis” (*sensu* HEA) of venom evolution has been blamed on inconsistencies in definitions of the word “venom” and the designation of certain species as “venomous”. In reality, the various definitions of “venom” differ little—the consensus is that venom is an actively delivered (e.g., via a bite or a string) secretion that functions (i.e., has been selected for the purpose of) in the subjugation of prey or the deterrence of predators/competitors [29,38,41]. A popular wording of this definition [42], sometimes considered to be more restricted than more recent formulations [43], introduces a point of confusion by including “subjugation *and/or* digestion *and/or* in defence”, which suggests that digestion is a distinct function of “venom”. This would greatly complicate matters, as oral secretions (e.g., human saliva) in general aid digestion. Here, we prefer the more restricted definition—whilst digestion is undoubtedly a function of the oral secretions of the lizards examined in the present study, that fact alone does not qualify the secretions as “venoms”. 

Confusion has thus been fueled less by differing definitions of “venom”, than by different applications of the term, and particularly its conflation with the term “venom system”. This confusion stems from the use of the two terms interchangeably in papers concerning Toxicofera going back at least as far as 2006 [23]. It must be stressed that taxa possessing a “venom system” are not necessarily “venomous”—in particular we have frequently referred to the venom system of iguanian lizards as “incipient” (e.g., [30] and see [41] for a discussion of the use of the term incipient in evolutionary contexts). So, the “Toxicofera hypothesis of venom evolution”, more properly considered (although this discussion does not constitute a formulation of such a hypothesis), concerns the early evolution of the venom *system*—i.e., the synapomorphy of the Toxicofera clade, which (as above) is the presence of protein-secreting oral glands (i.e., an incipient venom system) which may be considered exapted for the subsequent development of sophisticated venom delivery systems. 

In making a selectable contribution to the subjugation of prey, it is not necessary for a venom to be capable of rapidly killing or incapacitating the prey item. Venoms do not necessarily function as stand-alone prey subjugation mechanisms—they may be used in concert with physical means of subjugation. Thus, a venom system need only marginally increase a predator’s chances of successfully securing a meal, e.g., by slightly weakening a potential prey animal, making it easier to subdue physically [41]. When the effects of a varanid lizard venom are contrasted with those of the venom of a highly front-fanged snake such as a taipan as an argument against such lizards having a ‘true’ venom [43], the differences are indeed stark but this is a spurious comparison. This does not constitute a strong argument against the lizard being venomous, instead it only serves to illustrate the fact that venom systems exist in myriad states of development within Toxicofera, as they do within the advanced snakes themselves [39].

Criticisms of (some variants of) the Toxicofera hypothesis of venom evolution notwithstanding, it is clear that a core set of toxins are present in the venoms of all anguimorph lizards studied to date including CRiSP, kallikrein, B-type natriuretic peptides and type III phospholipase A_2_ (PLA_2_) [23,24,25,27,28,29,30,31,32,33,34]. It is an important point that the presence of a particular species of protein within a secretion does not demonstrate the function of that secretion, which is why the evolution of venom must be studied by considering the venom system as a whole—functional assays should be deployed, as well as considerations of associated anatomy (i.e., delivery mechanisms) and organismal feeding ecology. Some of the previously listed toxins are responsible for the hypotensive effect of intravenous injection of crude venom in rats [22,32,33], such as aortic smooth muscle relaxation by natriuretic peptides equipotent to forms recovered from venomous snakes [44]. In addition, kinin release from kininogen by kallikrein enzymes is another source of hypotensive effects resulting from lizard venoms [45]. In addition, it has also been previously noted that injection of rodents and birds with *V. griseus* venoms resulted in paralysis [46]. 

Reports of human envenomation by monitor lizards have been noted both in the literature and anecdotally. A bite report documented effects for a *V. griseus* bite to a zoo keeper that included symptoms similar to that of the bird and rodent studies: dizziness, muscular weakness and soreness in the extremities, facial and eye muscle pain, respiratory distress and pain (local and systemic) [47]. Additional reports of *V. griseus* bites to zoo keepers recorded similar symptoms including dysphagia, chest tightness, muscle soreness in the extremities, facial pain, dizziness, and difficulty walking [48]. In all the above *V. griseus* cases, the bite victims were experienced zoo keepers who did not view the bites with concern upon their occurrence. Vikrant and Verma (2014) reported a lethal bite by the related *V. bengalensis* that induced local pain, blood loss, as well as nausea, diaphoresis, dizziness, and breathlessness in the victim and eventually led to an acute kidney injury and cardiac arrest [49]. The actual culprit responsible for this bite has been questioned by clinical toxinologists, who considered it more likely to be a venomous snakebite [50]. However, it must be noted that this dissenting opinion by people who were not involved in the case did not provide any new and contradictory evidence. In contrast, a local wildlife officer who was present at the event confirmed the identity of the monitor lizard and the attending physicians documented the bite wounds as being inconsistent with the puncture wounds characteristic of bites by venomous snakes but rather consistent with the lacerations produced by monitor lizard bites (Vikrant personal communication). Thus, while this case is extraordinary, the possibility of dangerous bites from varanid lizards to humans, under exceptional circumstances, should not be discounted. Another case report described a bite by a juvenile *V. komodoensis* that led to faintness, prolonged bleeding and transient hypotension [51]. The authors attributed these effects to a vasovagal reaction despite noting themselves the similarity to reported *in vitro* effects of *V. komodoensis* venom [33] and with vasovagal reaction unable to explain the prolonged bleeding.

Anecdotally, a great many varanid lizard bites to biologists, zookeepers and amateur reptile enthusiasts have resulted in little that could be attributed to the action of toxins; however, some bite victims do report burning sensations, prolonged bleeding and inflammation disproportionate to the mechanical damage inflicted [10]. In addition to published cases, BGF has been contacted by keepers of varanid species who had symptomatic cases, sometimes with taxon-specific effects: 3 cases of *V. albigularis* reporting extreme muscle soreness and weakness lasting days, 4 cases of *V. kordensis* producing significant local swelling of the bitten finger and adjacent fingers, 5 cases of *V. varius* with pronounced bleeding lasting 3–4 h, a *V. salvadorii* bite with similar symptoms to that of *V. varius* and a *V. komodoensis* bite also producing apparent anticoagulant effects. These effects are consistent with the characterised venom chemistry of anguimorph lizards [23,29,32,33]. 

When compared with helodermatid lizard bites, which have received far more research attention than those of their varanoid cousins, a key point becomes evident: duration of contact. Typically, a helodermatid lizard will stay attached, chewing more venom into the bite site, while a monitor lizard is less likely to hold onto something that is not a food item but in some cases may hold on for up to a half hour when defensively biting. This likely leads to differences in the amount of oral fluids inoculated to the victim, with symptomatic human bites typically being those in which the varanid lizard chewed for a prolonged period of time. Interestingly, while feeding on large prey items, varanid lizards seem to have a tendency to shake it violently and chew vigorously until it is subdued, which may facilitate venom delivery as well as potentiating mechanical damage [52].

Varanoid lizards are characterised by a refined mandibular venom gland that is homologous with that of the helodermatid lizards [23,25,27,29,32,33,53]. Most anguimorph lizards have simple-structured mandibular venom glands, however, *Heloderma* and *Lanthanothus/Varanus* have independently evolved complex glands [32]. Both lineages derived their compartmentalised venom glands from the ancestral anguimorph lizard condition of an enlarged, mixed sero-mucous gland in which the protein- and mucous-secreting regions are not segregated into distinct glandular structures. In both cases, sophisticated structures with separated protein and mucous regions, a structured lumen for storing liquid venom, and a thick membranous cover, have evolved. Further to this, morphological as well as molecular evolutionary studies have indicated that these glands are homologous with the venom glands of snakes [23,25,27,29,32,33,53]. The fact that varanid lizards possess highly developed dental glands suggests that those glands in one way or another play an important role in their life. It has been shown with venomous snakes which switch to eggs or constriction as an alternate form of prey capture rapidly lose the functionality of their venom glands, with atrophying occurring in short periods of (evolutionary) time [25,29,54,55].

The evolution of a complex venom system is likely only possible under certain contingent circumstances—i.e., when both environmental conditions select for it and a species’ overall evolutionary trajectory facilitates it. For example, in Iguania the incipient venom glands never developed any significant complexity, probably due to the mainly insectivorous/herbivorous nature of these lizards. In addition, when a species evolves an alternative method of subduing prey that renders the venom system redundant, or switches to a diet with no need for subjugation (e.g., plants), the venom system often degrades [25,29,54,55]. The cost of venom production is presumably high enough to justify the presence of active secretory and delivery apparatuses only when it confers an evolutionary advantage [56]. It is notable, however, that in iguanian species which include a large quantity of vertebrates in their diet, the glands are larger and the protein-secreting region more developed (though we stress that this is not equivalent to them being ‘venomous’ per se but it is strongly suggestive of a functional role in predation) [29,30].

Previous work on helodermatid lizards has demonstrated a striking level of proteomic conservation within the venoms of this clade, with the same basic toxin groups present in similar quantities [27,57] despite the most recent common ancestor (MRCA) of the five *Heloderma* species existing ~15–20 million years ago [11]. While the venom glands of *Varanus* species have been compared transcriptomically, the only proteomic comparisons to-date were limited to SELDI mass spectrometry [23,32,33]. However a diversity of components have been functionally characterised from helodermatid and varanid venoms (Table 1), and since studies have demonstrated the complexity and medicinal potential of *Heloderma* venom [27,58,59,60,61,62,63,64] it is of interest to study the venom system of varanoid lizards in detail.

**Figure 1:**
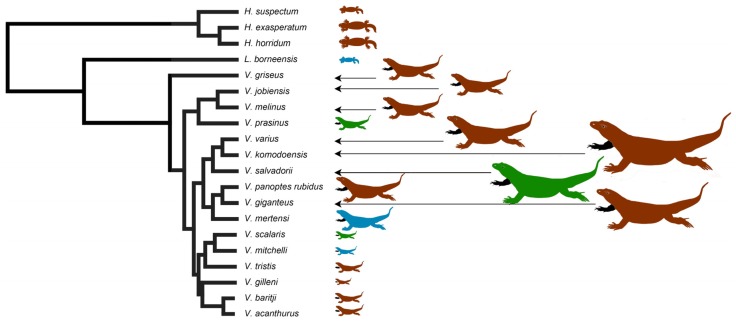
Organismal relationships, sizes and ecological niches occupied (blue = aquatic, brown = terrestrial, green = arboreal). Phylogeny based on [65,66,67]. Note that as it only includes species from this study, it excludes the other anguimorph lizards such as anguids that intervene between *Heloderma* and *Lanthantus/Varanus.* Red lineages are terrestrial, green are arboreal, and blue are aquatic.

Therefore, the aim of this study was to undertake a proteomic and functional comparison of varanoid lizard venoms in order to uncover patterns of venom evolution across the clade and to add to the body of knowledge regarding their controversial evolution. In view of treating the varanid lizard venom apparatus as an integrated system, the teeth of each species were also examined. Varanid lizards belong to the lizard clade Anguimorpha that also contains anguid and helodermatid lizards [13,14,67], of which the gila monster (*Heloderma suspectum*) and Komodo dragon (*V. komodoensis*) are the most well-known species. *Varanus* is the sole extant genus in the family Varanidae and its closest extant relative is *Lanthanotus borneensis* (Borneo earless monitor-Lanthanotidae), also included in this study. Together Varanidae and Lanthanotidae (along with numerous extinct taxa) comprise the superfamily Varanoidea, of which *Heloderma* was previously also considered a member. Lizards in the genus *Varanus* are squamate reptiles with total body length ranging from 23 cm for adult *V. brevicauda* to over 3 m for *V. komodoensis* (Figure 1). 

## 2. Results

### 2.1. Teeth Scanning Electron Microscopy

**Figure 2:**
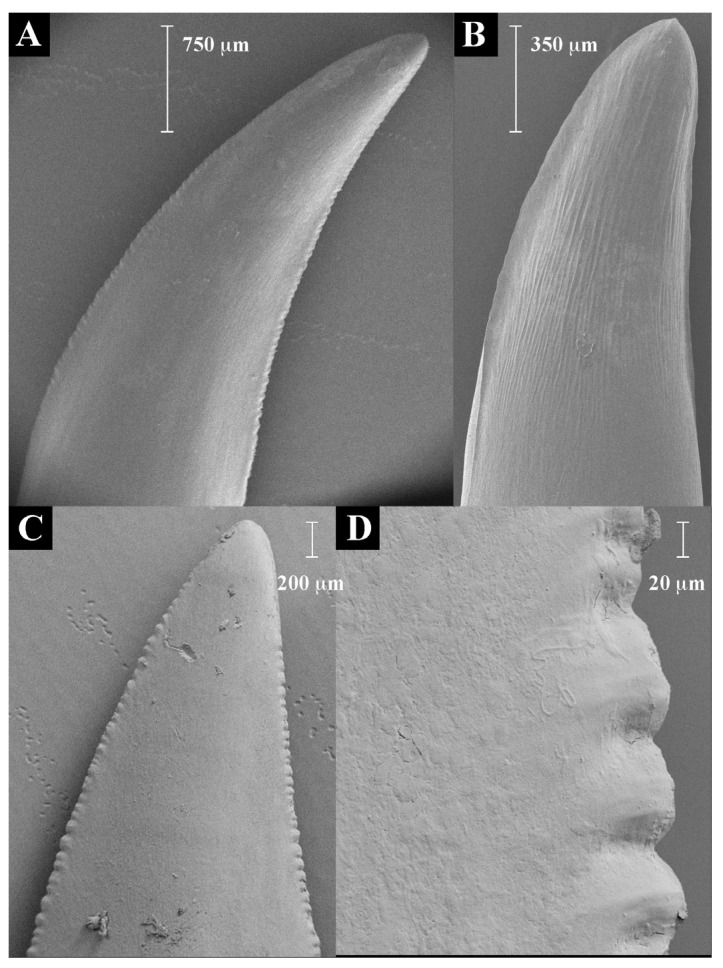
Scanning electron microscopy showing the (**A**) plesiomorphic moderate/medium anterior and posterior serrations such as in *V. giganteus*, (**B**) the derived non-serrated condition as in *V. mertensi*, (**B**) or (**C**,**D**) derived deep anterior and posterior serrations as in *V. komodoensis*.

**Figure 3:**
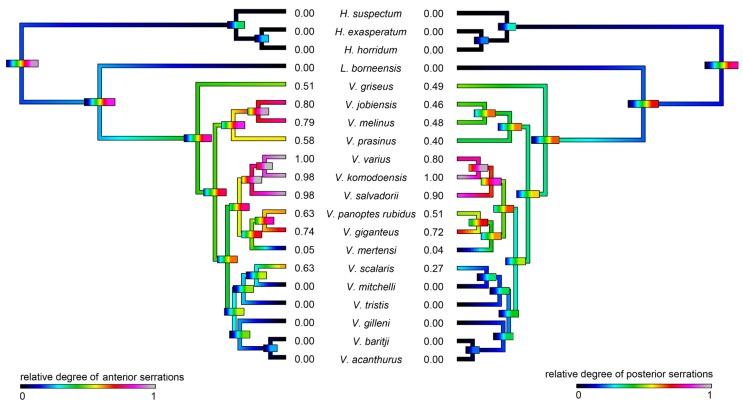
Ancestral state reconstructions over branches for anterior teeth serrations and posterior teeth serrations inhibition where warmer colours represent more serrations. Bars indicate 95% confidence intervals for the estimate at each node. Note that due to the high dynamicity of venom evolution these quickly become broad as you move down the tree. Phylogeny follows [65,66,67].

In the 20 species of varanoid lizard examined (Figure 1), the relative degree of serration (Figure 2 and Figure 3) in the anterior vs posterior edges of teeth were reciprocally strongly and positively related (PGLS: *t* = 28.1998, *p* = 2.22 × 10^−16^). Differential evolution was evident in the stronger serrations of the *V. varius* clade, while conversely the clade of *V. acanthurus*, *V. baritji*, *V. gilleni*, *V. mitchelli*, *V. scalaris*, and *V. tristis* were typified by a secondary loss of serrations, which the exception of *V. scalaris* which re-evolved serrations within this clade.

### 2.2. Proteomics Studies

**Figure 4:**
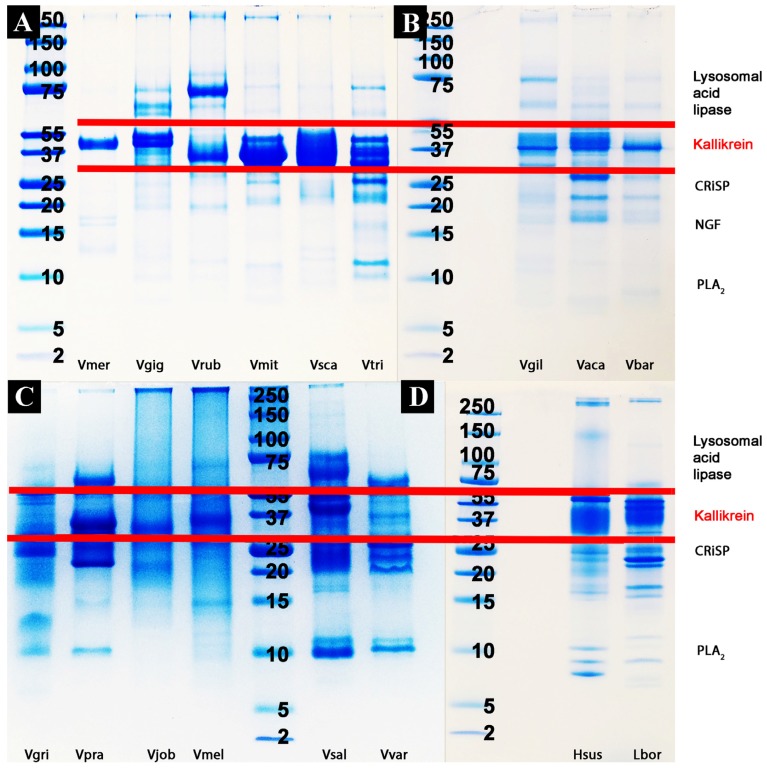
1D gel comparisons: (**A**) Vmer = *V. mertensi*, Vgig = *V. giganteus*, Vrub = *V. panoptes rubidus*, Vmit = *V. mitchelli*, Vsca = *V. scalaris*, Vtri = *V. tristis*; (**B**) Vgil = *V. gilleni*, Vaca = *V. acanthurus*, Vbar = *V. baritji*; (**C**) Vgri = *V. griseus*, Vpra = *V. prasinus*, Vjob = *V. jobiensis*, Vmel = *V. melinus*, Vsal = *V. salvadorii*, Vvar = *V. varius*; and (**D**) Hsus = *H. suspectum*, Lbor = *L. borneensis.* Molecular markers are shown in kDa.

**Figure 5:**
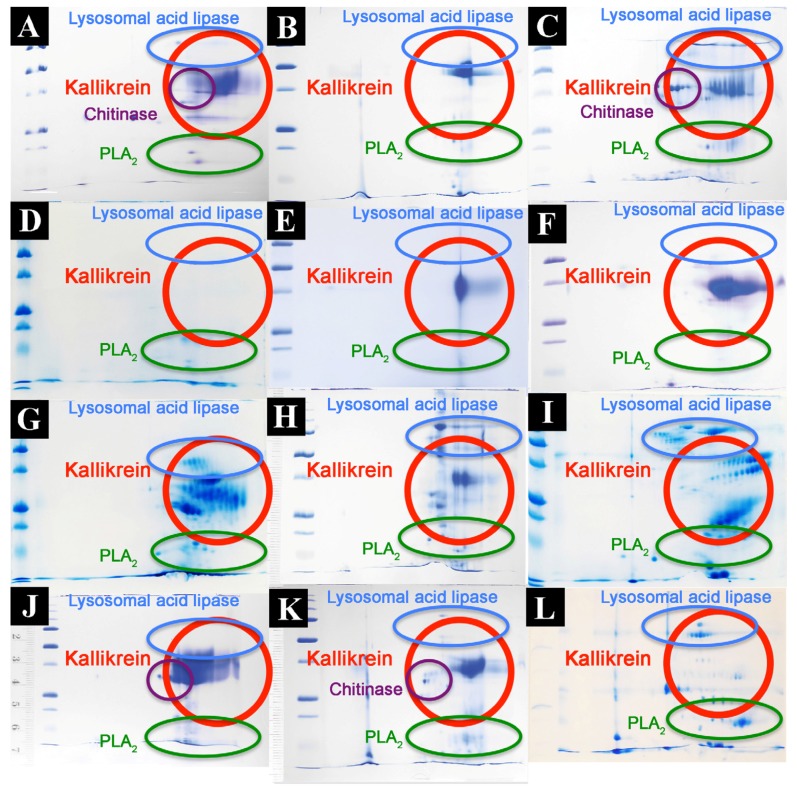
Varanid venom 2D gel comparisons showing the relative kallikrein presence. (**A**) *Varanus acanthurus*, (**B**) *Varanus giganteus*, (**C**) *Varanus gilleni*, (**D**) *Varanus griseus*, (**E**) *Varanus mertensi*, (**F**) *Varanus mitchelli*, (**G**) *Varanus prasinus*, (**H**) *Varanus rubidus*, (**I**) *Varanus salvadorii*, (**J**) *Varanus scalaris*, (**K**) *Varanus tristis*, and (**L**) *Varanus varius*.

Proteomic analyses revealed the venoms to contain a large diversity of components, with kallikrein predominating in all species except *V. griseus* and *V. varius*, suggesting it plays a lesser role in these species (Figure 4 and Figure 5, Table 2). Full MS/MS data are available in Supplementary Tables S1. 2D gels show that the kallikrein toxins possess significant diversity in isoelectric points and molecular weight diversity (Figure 5), ranging from the narrow but dense spots of *V. giganteus* (Figure 5B) and *V. mertensi* (Figure 5E) through to the evolution of differential forms in *V. prasinus* (Figure 5G) and extending to poorly staining low levels in *V. griseus* (Figure 5D) and *V. varius* (Figure 5L). There were significant kallikrein expression variations in closely related taxa, ranging from broad isoelectric point variation in *V. scalaris* (Figure 5J) relative to *V. tristis* (Figure 5K), through to downregulation in *V. varius* (Figure 5L) relative to *V. salvadorii* (Figure 5I). 

*L. borneensis* venom displayed a 1D pattern more similar to the putatively ancestral type shared with *Heloderma* species (Figure 4) [57]. In contrast, the varanid 1D gels (Figure 4) revealed extensive variation is detectable outside regions of the gel corresponding to kallikrein (which remains relatively invariant across species). Chitinase/chitotriosidase were present in all the venoms of the dwarf species, but virtually absent from those of larger species (Figure 4 and Figure 5, Table 2).

### 2.3. Molecular Evolution

#### 2.3.1. Kallikrein Molecular Evolution

**Figure 6:**
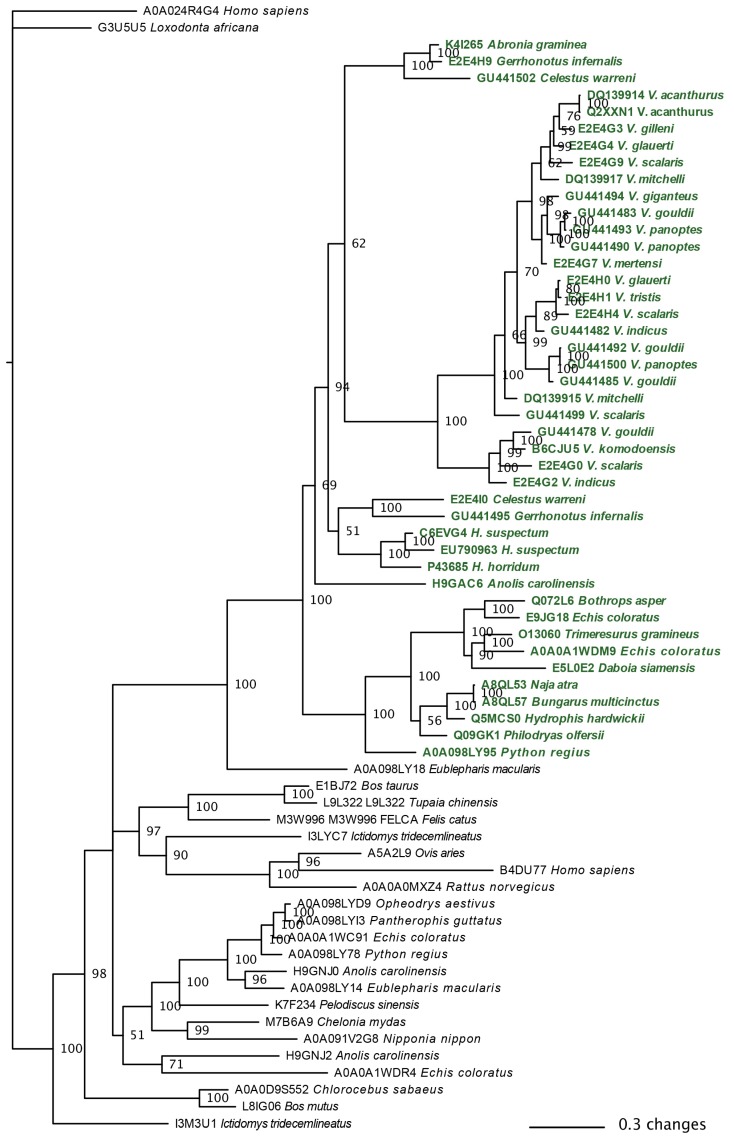
Molecular phylogenetic relationships of anguimorph lizard kallikrein enzymes. Numbers at node indicate probability. A monophyletic clade of toxicoferan mandibular and maxillary gland sequences is shown in green.

The molecular phylogeny of the available anguimorph kallikrein sequences showed evidence of gene duplication and diversification (Figure 6). In contrast to a previous study challenging kallikrein as a shared toxin type between snakes and lizard venoms based on the non-monophyly of toxicoferan oral gland sequences relative to non-toxicoferan oral gland and body tissue forms [40], we recovered all toxicoferan gland sequences as a monophyletic group relative to sequences from body tissues or from non-toxicoferan species. As the authors of that study did not provide their alignments or run files despite repeated requests by us, we cannot ascertain the cause of the discrepancy as due to either alignment issues with that previous study or differential phylogenetic methods. Regardless, our tree provides high node support (Figure 6) in contrast to the unresolved polytomies in Supplementary Figure S19 of the Hargreaves et al. study [40].

**Figure 7:**
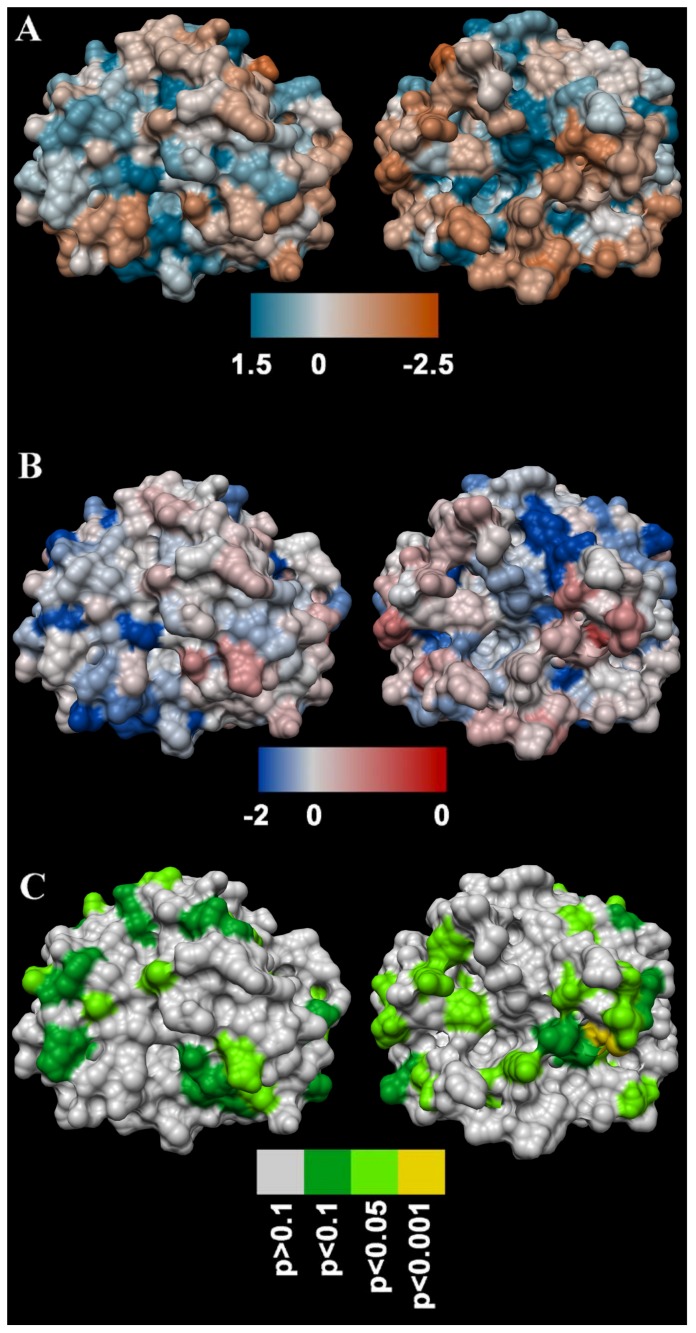
Three dimensional structure of a kallikrein toxin coloured according to (**A**) AL2CO amino acid conservation score (conserved sites in teal and variable sites in orange), (**B**) FUBAR strength of persistent selection (sites under purifying selection in blue and sites under diversifying selection in red), and (**C**) MEME significance levels for episodes of diversifying selection during the evolution of the toxin family (moderately significant sites in dark green, highly significant sites in light green, and extremely significant sites in yellow).

Molecular modelling demonstrated the overall dN/dS value for anguimorph kallikrein toxins for which nucleotide data are publicly available was 0.80, indicating that the protein as a whole has been subject to neutral or weak purifying selection. Under negative or purifying selection, less “fit” nonsynonymous substitutions accumulate more slowly than synonymous substitutions, and under diversifying or positive selection, the converse is true. However, the FUBAR (Fast Unconstrained Bayesian AppRoximation) and MEME (Mixed Effects Model of Evolution) methods detected a number of individual sites on the toxin surface (Figure 7) that are likely to have been subject to diversifying selection. This suggests that these sites may be important in the coevolutionary arms race between anguimorphs and their prey and may well play a role in the function of the toxins [87]. It should be noted that the analyses were conducted without the inclusion of a *H. horridum* sequence originally stated containing an insert characteristic only of snake venom forms [84] but which was subsequently shown to be erroneous [32]. However in the intervening time, this sequencing error led a study to conclude that convergent evolution had occurred between lizard and shrew toxin forms [88] and has been repeatedly cited as evidence for such convergence [89,90,91,92,93,94,95,96,97,98,99,100,101,102,103,104,105,106,107,108,109,110,111,112].

Outside of the core kallikrein enzyme, other widely expressed protein types included CRiSP, lysosomal acid lipase and PLA_2_. However, in contrast to the in general strong presence of kallikrein, these were more variable. Shotgun MS/MS revealed the presence of additional toxin types not discernible by gel-based methods such as the small natriuretic peptides (Table 2). 

#### 2.3.2. Lysosomal Acid Lipase Molecular Phylogeny

**Figure 8:**
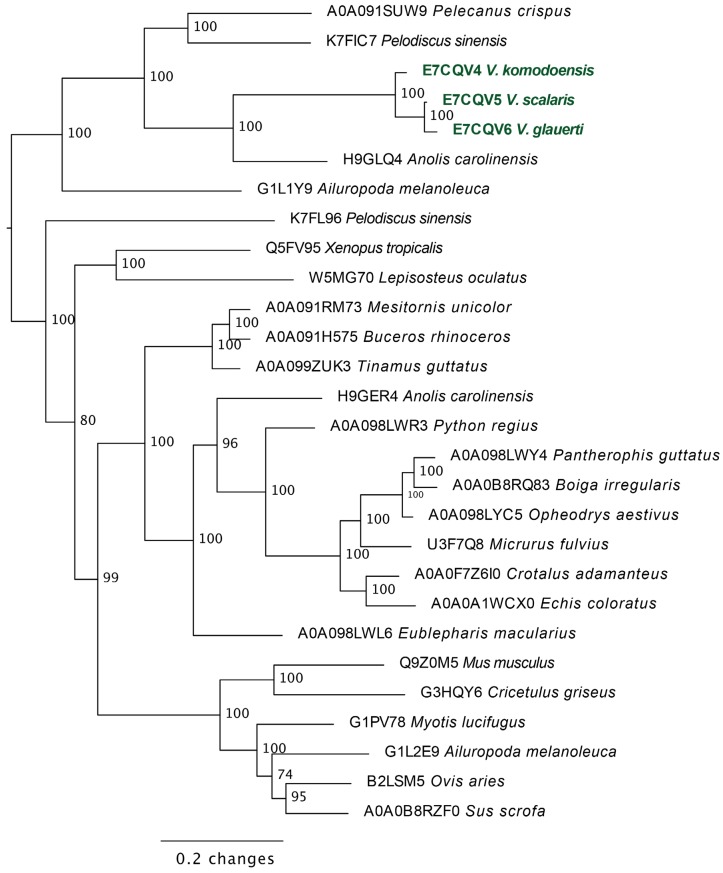
Molecular phylogenetic relationships of anguimorph lizard lysosomal acid lipase enzymes relative to other sequences. Varanid venom gland sequences are shown in green.

Molecular phylogeny revealed the anguimorph lizard lysosomal acid lipase sequences to form a clade distinct from those sequenced from snake venom glands (Figure 8).

### 2.4. Bioactivity Testing

**Figure 9:**
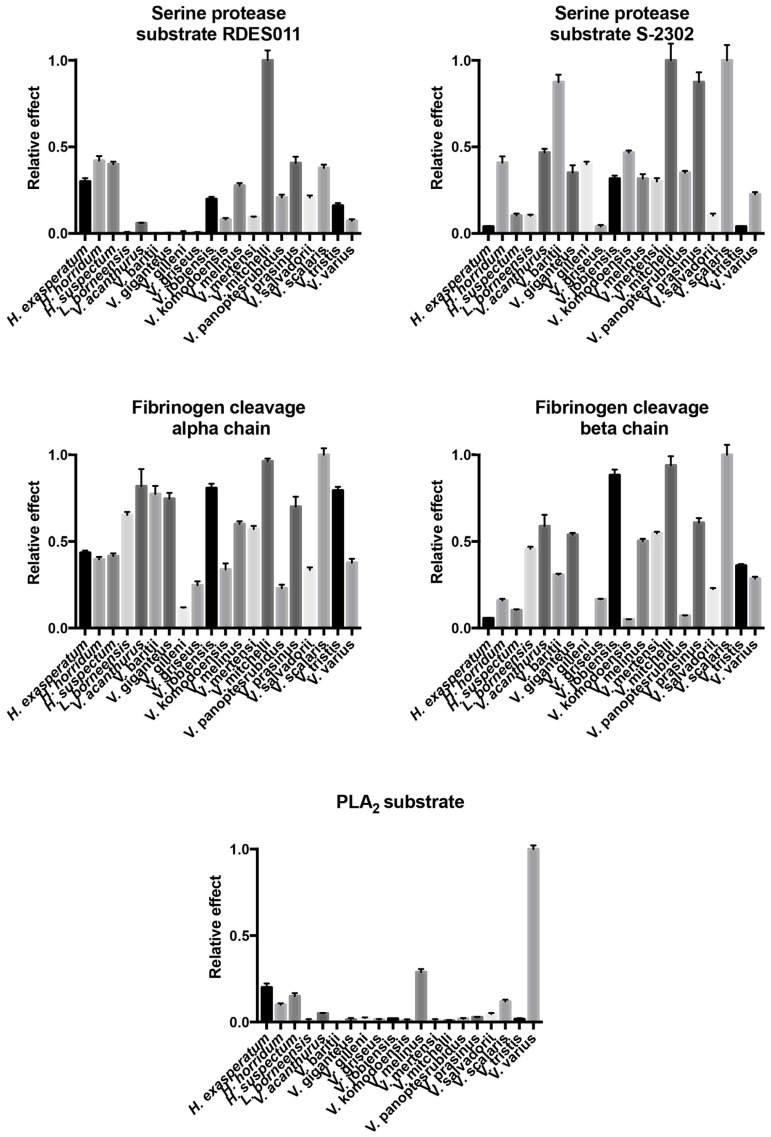
Activity levels for the bioactivity assays shown as *n* = 3 means with standard deviation error bars.

Bioactivity testing revealed dynamic variation in activities between and within each assay type with statistical significance between species for each assay (Figure 9, Table 3). Raw values are shown in Supplementary Table S2 and tests for significance are shown in the Supplementary Tables S3.

#### 2.4.1. Kallikrein Enzymatic Activity upon Fluorescent Substrates

**Figure 10:**
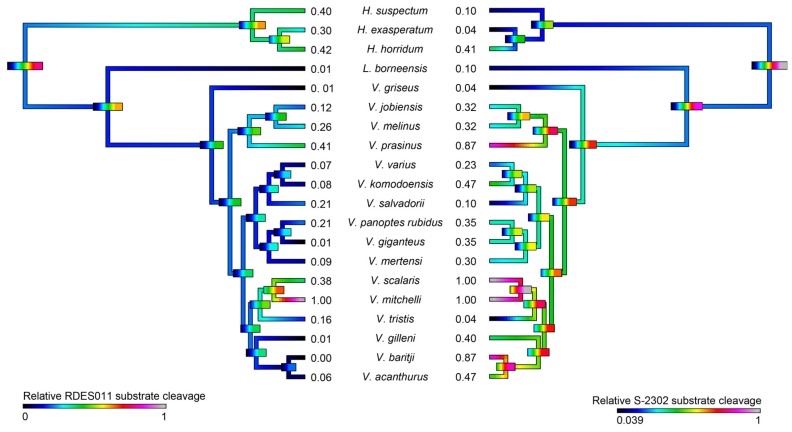
Ancestral state reconstruction of serine protease substrate activity, where warmer colours represent more potent substrate cleavage. Bars indicate 95% confidence intervals for the estimate at each node. Note that due to the high dynamicity of venom evolution the ranges quickly become broad as one moves down the tree. Phylogeny follows [65,66,67].

Consistent with the evidence for kallikrein molecular diversification, the ability of the venoms to cleave serine protease substrates varied significantly among the species studied but with phylogenetic patterns evident (Figure 10). In addition, some species’ venoms were potent on one substrate but not the other, with *V. mitchelli* the most potent upon both substrates. 

#### 2.4.2. Fibrinogen Cleavage Gels

**Figure 11:**
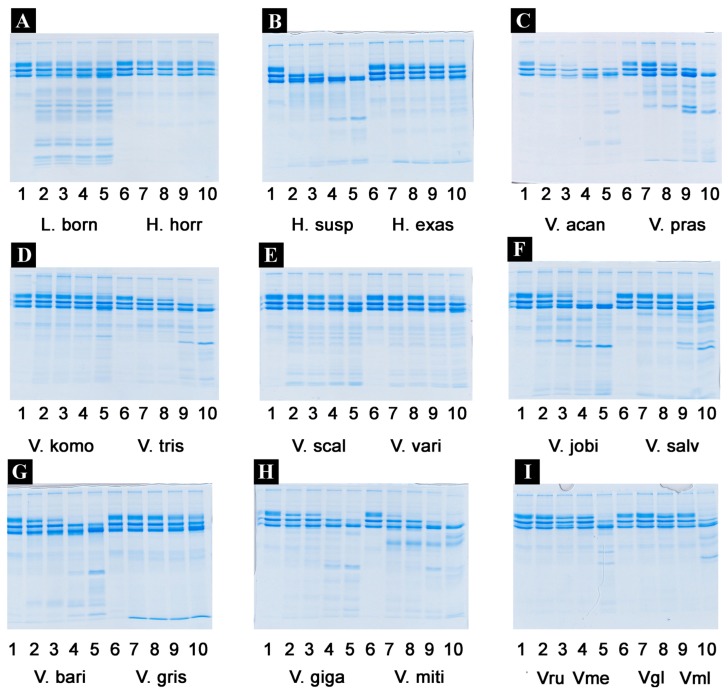
Fibrinogen cleavage over time. For **gels** (**A**–**H**) lanes 1&6 = time zero, lanes 2&7 = 1 min incubation, lanes 3&8 = 5 min incubation, lanes 4&9 = 20 min incubation, and lanes 5&10 = 60 min incubation. For **gel** (**I**) lanes 1&6 = time zero, lanes 2, 4, 7 & 9 = 5 min incubation, and lanes 3, 5, 8, & 10 = 60 min incubation. L. born = *L. borneensis*, H. exas = *H. exasperatum*, H. horr = *H. horridum*, H. susp = *H. suspectum*, V. acan = *V. acanthurus*, V. bari = *V. baritji*, V. giga = *V. giganteus*, Vgl = *V. gilleni*, V. gris = *V. griseus*, V. komo = *V. komodoensis*, V. jobi = *V. jobiensis*, V. miti = *V. mitchelli*, VmL = *V. melinus*, Vme = *V. mertensi*, V. pras = *V. prasinus*, Vru = *V. panoptes rubidus*, V. scal = *V. scalaris*, V. salv = *V. salvadorii*, V. tris = *V. tristis*, and V. vari = *V. varius*. Gels were run in triplicate, with the patterns congruent between replicates.

**Figure 12:**
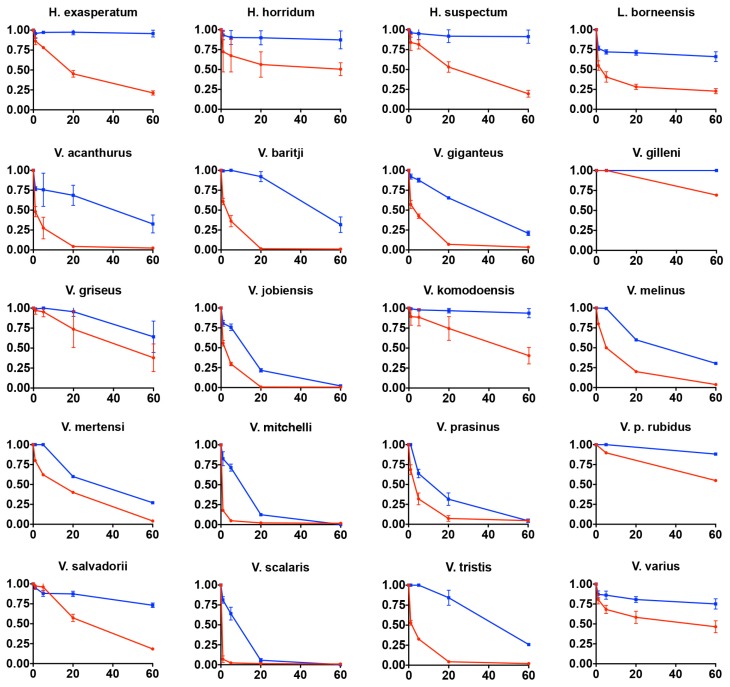
Relative cleavage of alpha (red) or beta (blue) chains of fibrinogen. X-axis is time (min) and y-axis is percentage of intact chain remaining. Error bars indicate *n* = 3 standard deviation.

**Figure 13:**
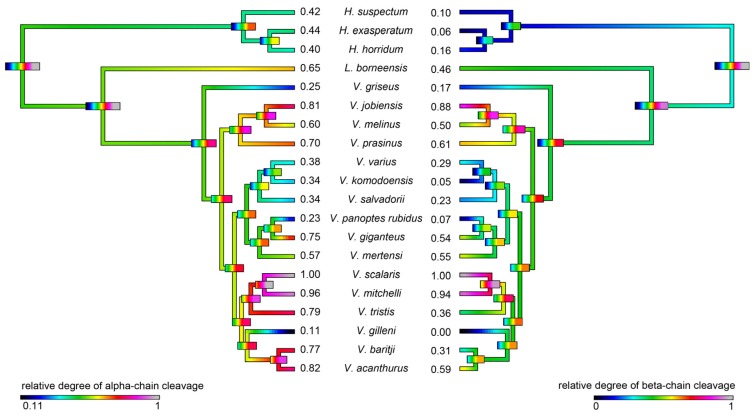
Ancestral state reconstructions over branches for destructive cleavage of fibrinogen alpha and beta chains. Bars indicate 95% confidence intervals for the estimate at each node. Note that due to the high dynamicity of venom evolution these quickly become broad as one moves down the tree. Phylogeny follows [65,66,67].

**Figure 14:**
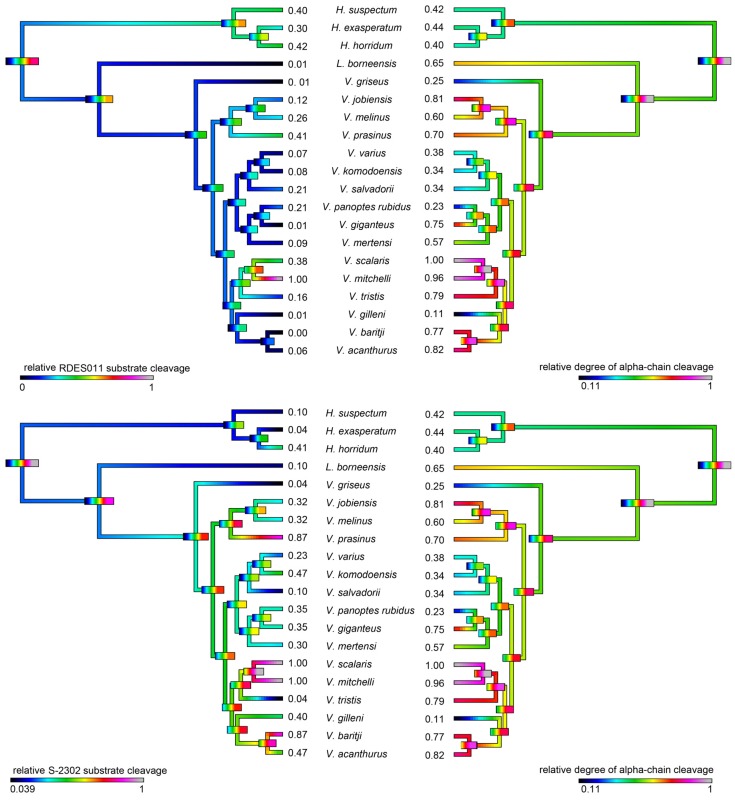
Ancestral state reconstructions over branches comparing substrate consumption relative to alpha chain destruction. Bars indicate 95% confidence intervals for the estimate at each node. Note that due to the high dynamicity of venom evolution these quickly become broad as one moves down the tree. Phylogeny follows [65,66,67].

**Figure 15:**
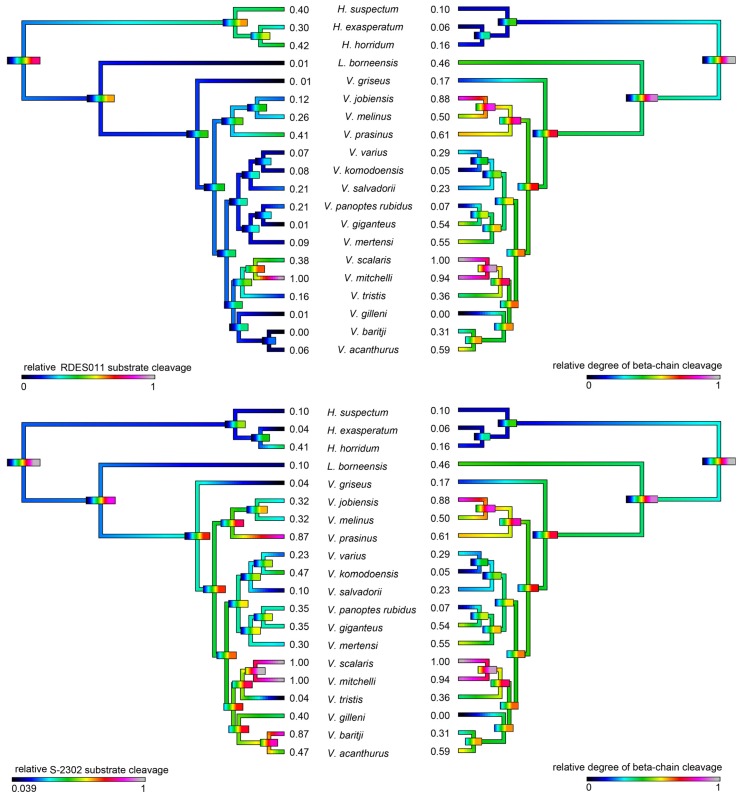
Ancestral state reconstructions over branches comparing substrate consumption relative to beta chain destruction. Bars indicate 95% confidence intervals for the estimate at each node. Note that due to the high dynamicity of venom evolution these quickly become broad as one moves down the tree. Phylogeny follows [65,66,67].

As kallikrein toxins isolated from *Heloderma* venoms have been shown to exert non-clotting, destructive cleavage of fibrinogen [79,85], we investigated the relative time-dependent actions of venoms in this study for such actions (Figure 11, Figure 12 and Figure 13). Intriguingly, differences in cleavage products indicated differential cleavage sites. A consistent pattern emerged in which all venoms displayed some ability to cleave the Aα chain, while the Bβ chain was cleaved more slowly and only fully destroyed by the most potent Aα chain acting venoms. High relative Aα chain activity predicted correspondingly high relative Bβ chain activity (PGLS: *t* = 7.3532, *p* = 7.969 × 10^−7^). Aα chain cleavage was well predicted by activity upon the substrate S-2302 (PGLS: *t* = 2.8968, *p* = 0.009611, Figure 14) as was Bβ chain activity (PGLS: *t* = 2.8459, *p* = 0.01073, Figure 15). However, the γ chain was untouched even in species which fully consumed the Bβ chain. 

#### 2.4.3. Phospholipase A_2_ Enzymatic Activity upon Fluorescent Substrate

**Figure 16:**
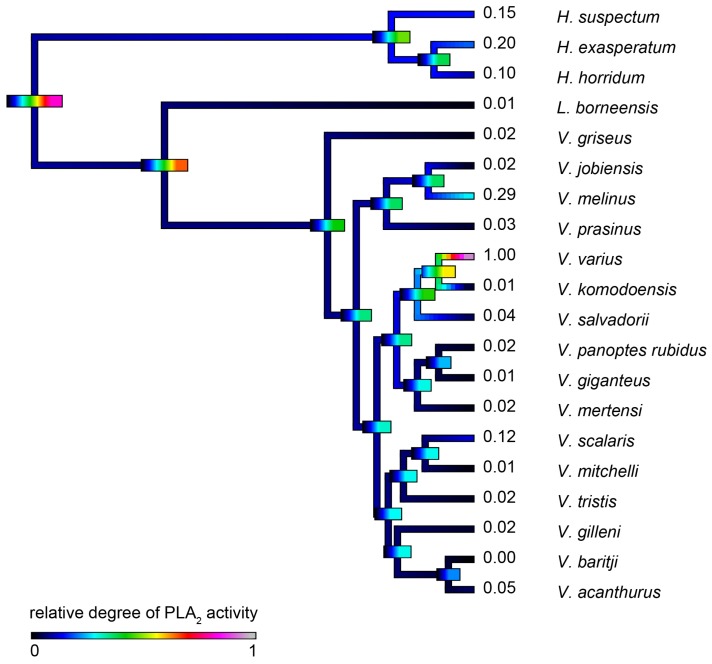
Ancestral state reconstructions over branches for PLA_2_ enzyme substrate consumption where warmer colours represent more fluorescence production. Bars indicate 95% confidence intervals for the estimate at each node. Note that due to the high dynamicity of venom evolution these quickly become broad as one moves down the tree. Phylogeny follows [65,66,67].

*V. varius* venom had a dramatically higher level of PLA_2_ enzymatic activity than other species tested (Figure 16), and thus obscured that other species displayed significant levels of PLA_2_ activity consistent with the previous transcriptomic, proteomic or functional evidence for the presence of this enzyme type [23,32,86].

#### 2.4.4. Rat Ileum Contraction Organ Bath Assay

**Figure 17:**
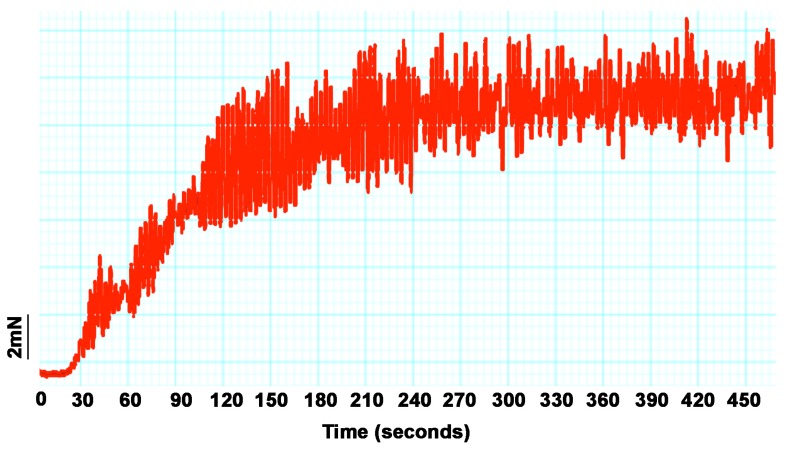
Change in rat ileum smooth muscle contractility after administration of crude 50 μg/mL *V. varius* venom. On the Y-axis is the pulling force in relative units; on the X-axis is time. Experiment was run in triplicate and results were congruent between replicates.

*V. varius* venom demonstrated strong smooth muscle contracting activity (Figure 17) consistent with the previously documented presence of AVIT and cholecystoxin peptides and the demonstrated action for snake venom homologues of this toxin type [32,33,113]. Due to lack of sufficient venom, other species were not tested.

## 3. Discussion

The venoms of varanoid lizards remain understudied in evolutionary toxinology; however, multiple sources of evidence point to the adaptive evolution of venom in varanoid lizards. The present study revealed the differential complexity of anguimorph lizard venoms across a wide taxonomical range (Figure 1) and considered its evolutionary context as part of a combined predatory arsenal. 

Prior to this study, tooth form and function have been reported for very few species of varanid lizards [1,114,115] and no study has compared tooth morphology and function across many species. The presence of serrations can be important indicators of tooth function in reptiles. Serrated teeth cut compliant material by presenting less contact area with the material, and thus the applied force at each point of contact is relatively greater. Serrations also act to trap and cut sections of material with a ‘bite and slice’ mechanism allowing a sliding force to be transferred into a cutting force [116]. Serrations have been reported for the teeth of *V. komodoensis*, *V. salvator*, and *V. varius* [1,114,115]. Juvenile *V. niloticus* also appear to bear serrations on the anterior and posterior edges, but these are lost in the adult form, to become smooth and blunt, reflecting a change in diet from insectivorous to molluscivorous in adults [117]. 

Slightly serrated teeth appear to be the plesiomorphic condition of varanid lizards (Figure 2 and Figure 3). Significant diversification from the inferred plesiotypic tooth state was evident in two groups: the *V. varius* clade and members of the *Odatria* subgenus of dwarf monitors (*V. acanthurus*, *V. baritji*, *V. gilleni*, *V. mitchelli*, *V. scalaris*, and *V. tristis*) (Figure 3). Each of these groups also possesses morphological features and predatory strategies characteristic of the particular clade. *V. komodoensis*, *V. salvadorii* and *V. varius* had the most pronounced serrations, consistent with these species typically feeding on thick-skinned large mammalian prey that they dismember. Thus while there were not any evident direct links to venom chemical composition and serrations, there was for prey dismemberment strategies. The aquatic lineage *V. mertensi* thus represents a secondary loss of serrations. The dwarf species (*Odatria*) have also secondarily lost the serrations, with the sole exception within the clade being the arboreal lineage *V. scalaris* which has subsequently re-evolved serrations. The reasons for the unique (amongst *Odatria*) evolution of serrated teeth in *V. scalaris* may be linked to the inclusion of large, chitinous insects in its diet, which may require dismemberment before ingestion, or may be linked to active defence of territory from conspecifics [118]. In addition, this species predates upon the large treefrog *Litoria splendida* in its range, which it dismembers, eating only the legs and avoiding the large toxin-secreting parotid glands (Fry personal observations). 

The extreme proteomic variability of varanid venoms, in contrast to the highly conserved nature of helodermatid venoms (Figure 4 and Figure 5), taken in concert with the evidence of duplication and diversification of toxin genes such as kallikrein within *Varanus* (Figure 6 and Figure 7) and other toxin types analysed previously [119]), is suggestive of adaptive evolution as a generator of toxin diversity in *Varanus* venoms. 

The venoms studied contained myriad toxins, although with the exception of kallikrein none of those components were consistently highly expressed in the venom. Thus, a kallikrein-dominated venom is inferred to be reflective of the condition of the venom system of the anguimorph lizard MRCA. These functional and proteomic results are congruent with previous transcriptome studies which recovered kallikrein as the dominant toxin type [23,27,32,33]. Kallikrein enzymes have clearly undergone significant structural and functional diversification within the clade. There was evidence of gene duplication (Figure 6) and diversification of the molecular surface biochemistry (Figure 7). The cleavage of the two fluorescent substrates were incongruent with each other, thus indicating variations in enzyme specificity (Figure 9A,B and Figure 10). Similarly the actions upon fibrinogen produced variations in degradation products even when the same chains were targeted, also indicative of diversification of enzyme specificity (Figure 11, Figure 12 and Figure 13). Thus, there is strong evidence for adaptive evolution of kallikrein toxins in the venoms of anguimorph lizards. 

The venoms of larger species of monitor were generally seen to be more complex than that of their smaller congeners (Figure 4 and Figure 5), which may be explained by the broader dietary range in larger species, both of the adult animals and across the life history. Thus, while multifunctional kallikrein enzyme toxins form the venom core, there has been additional diversification in the chemical composition of the venoms. As dietary studies of *V. varius* show [120], it has the broadest possible dietary range, feeding on everything from small invertebrates and eggs to medium-sized mammals, most likely experiencing ontogenetic niche shifts, which further necessitate adaptation to different prey items. This species also has the most distinct venom of all those in this study, with a secondary downregulation of kallikrein paralleled by an upregulation of other toxins which produce effects ranging from muscle contraction in this study (Figure 15) through to platelet-inhibition mediated anticoagulation shown previously [33]. 

Smaller species (members of the *Odatria* subgenus), on the other hand, predominantly feed on lizards and insects throughout their life [121]. These species retain potent fibrinogen destruction activities in their kallikrein toxins with the exception of *V. gilleni* (Figure 10, Figure 11 and Figure 12), the smallest species studied. It is notable that *V. gilleni* had the least amount of fibrinogen-targeting activity despite being rich in kallikrein toxins and having a stronger action upon substrate S-2302 than some other species which displayed stronger fibrinogen destruction activity (Figure 8). Other species were also rich in kallikrein on the gels but without corresponding activity upon either of the substrates tested. The kallikrein action in these species may instead be linked to high kininogen cleavage activity, with liberated kinins causing a rapid hypotensive effect in envenomed prey/predators. As this was beyond the scope of this study, future work on anguimorph lizard venoms should include assaying the relative release of kinins from kininogen by these venoms and the role this plays in the induction of hypotension. 

Previous transcriptome studies revealed the presence of kallikrein transcripts as the dominant types in venom gland transcriptome of varanid lizards and snakes, and these toxins have been inferred as present in the oral glands of the Toxicofera MRCA [23,24,27,28,29,30,32,33,34]. Characterised toxicoferan venom kallikreins induce fibrinogen depletion (Figure 11, Figure 12 and Figure 13) in the prey organism and thus promote prolonged bleeding [24] as well as induction of hypotension through the release of kinins from kininogen [45]. Predators likely benefit from inducing blood loss or altering the blood pressure of their prey, as this will increase the chance of successful subjugation by weakening the prey. Previous work has shown that the Type III PLA_2_ in *V. varius* venom block platelet aggregation, thus interfering with blood clotting via the same pathway as do homologous toxins in the venom of *Heloderma* species [33]. This species also had extremely high levels of PLA_2_ activity in comparison to all other species (Figure 16). In the case of some monitor lizards, in particular the larger species such as *V. varius* or *V. komodoensis*, this type of toxic action might be beneficial even if a prey manages to escape the initial attack but succumbs to blood loss or shock in the aftermath. Field studies on *V. komodoensis* have indeed shown such post-bite mortality in a significant percentage (~20%) of prey animals [33,122].

Venom may have functions other than aiding in subjugation of prey. For instance, venom may help in antipredator defence or in intraspecific competition, and may have additional roles in digestion, by providing additional enzymatic activity, or in maintaining oral health by including antimicrobial agents [29,30,41,123,124]. Indeed, venoms are likely to be multifunctional in many (perhaps most) species with either the same or different toxins facilitating multiple ecological functions [26,29,30,41,123,125,126], and the range of roles venom may play in varanids and their relative importance remain uncertain. In addition to possible roles in predation, defence or competitor deterrence, the role of varanid oral secretions in aiding digestion (a typical function of vertebrate oral secretions) has previously been put forward as a testable hypothesis [123] and our data appear to support this additional role, at least for certain species. While the venoms of larger species showed evidence of derived activities, so did those of the dwarf species. Dwarf monitors, unique to Australia, grow no larger than 600 mm SVL—chitinase enzymes are likely helpful in the digestion of the thick exoskeletons of the arthropods that are their primary food source. 

One salient hypothesis concerning effects of varanid lizard bites, such as hyperalgesia, inflammation and prolonged bleeding, is that the toxins (particularly those of dwarf species that are frequently predated upon by pythons) responsible for these effects may also serve defensive roles [10,123]. While kallikrein enzymes produce painful swelling, hyperalgesic cramping is a feature of the AVIT toxins type, which is also consistent with the strong muscular contraction observed for *V. varius* venom (Figure 17). Painful cramping would also aid in prey subjugation through loss of mobility. Another possible function of the secretions is intraspecific competition, particularly male-male combat, similar to the function of venom in platypus ecology [124]. In both such cases, rapid induction of hypotension by kinin release from kininogen would also be beneficial, thus reinforcing the multifunctional use of this functionally flexible enzyme type.

The presence of kallikrein alongside chitinase in the venoms of *Odatria* suggests that these secretions may serve multiple functional roles for these lizards—multifunctionality of toxic secretions is common in venomous organisms, and where venom is an oral secretion it often preserves its more primitive role in digestion [41]. Some components recovered in the study are very likely to be an adaptation for arthropod-based diets, which strengthens the point that venom glands in varanoid lizards likely have more than a single function. That is, in addition to its potential role in prey subjugation, defence, or intraspecific competition (the classic roles of “venom” *sensu stricto* [41]), varanoid lizard oral secretions may potentially aid in digestion.

According to our results, varanoid lizard venom is largely based on kallikrein toxins that previous studies have shown to be homologous with those present in the venom of advanced snakes [23,24,27,28,29,30,32,33,34]. Additional components are present in various species with profile complexity seemingly being a function of size and habitat where larger monitors possess the most complex venom and smaller or aquatic species the least. The high level of variability of varanid venoms relative to the high levels of conservation in helodermatid lizards, points to active evolution under selection pressure. Components such as lysosomal acid lipase (Figure 8) are functionally uncharacterised but underscore the rich biodiscovery potential of lizard venoms. As we were able to examine the venoms of only 16 of the more than 60 extant species of varanid lizard, this study is clearly not a comprehensive investigation of the evolution of venom in *Varanus*, and future studies are very likely to provide additional novel insights. For example, it will be fruitful to investigate the venom profile of *V. salvator*, a large but predominantly aquatic lizard, and frugivorous species such as *V. olivaceus*. Nevertheless, we note that turning away from this fruitful area of research by denying the biochemical reality of lizard venom will hinder progress in this fascinating area. For example, because of their chain-selective fibrinogen-degrading activity, kallikrein enzymes from snake venom have been used as anticoagulant treatment of stroke, heart attack, and deep-vein thrombosis [85,127]. The functional diversity of such enzymes in the lizard venoms in this study underscores the rich biodiscovery and therapeutic potential of these novel natural products.

## 4. Materials and Methods

### 4.1. Species Studied

Australian samples were previously collected at the same time as a transcriptome study [32] under University of Melbourne (2005) approval UM0706247 as part of the long-term cryogenic collection of the Venom Evolution Laboratory, while non-Australian samples were supplied by Alphabiotoxine. Species studied were *H. exasperatum* (captive bred specimens of unknown founder stock), *H. horridum* (captive bred specimens of unknown founder stock), *H. suspectum* (captive bred specimens of unknown founder stock), *Lanthanothus borneensis* (captive bred specimens of unknown founder stock), *Varanus acanthurus* (Newman, WA, Australia), *V. baritji* (Adelaide River, NT, Australia), *V. giganteus* (Sandstone, WA, Australia), *V. gilleni* (captive bred specimens of unknown founder stock), *V. griseus* (captive bred specimen of unknown founder stock), *V. jobiensis* (captive bred specimens of unknown founder stock), *V. melinus* (captive bred specimens of unknown founder stock), *V. komodoensis* (Singapore Zoo captive bred specimens of unknown founder stock), *V. melinus* (captive bred specimens of unknown founder stock), *V. mertensi* (Kununurra, WA, Australia), *V. prasinus* (captive bred specimen of unknown founder stock), *V. panoptes rubidus* (Sandstone, WA, Australia), *V. mitchelli* (Kununurra, WA, Australia), *V. prasinus* (captive bred specimens of unknown founder stock), *V. melinus* (captive bred specimens of unknown founder stock), *V. scalaris* (Kununurra, WA, Australia), *V. salvadorii* (captive bred specimens of unknown founder stock), *V. tristis* (captive bred specimens of unknown founder stock), and *V. varius* (Mallacoota, VIC, Australia). 3 adult specimens for each species were pooled to minimize the effects individual variation. In order to remove mucous, all samples were filtered through 0.2 micron syringe filters prior to lyophilisation.

### 4.2. Scanning Electron Microscopy

Teeth were obtained from museum or frozen specimens and dissected out of both the top and bottom jaw. The largest tooth available (usually the ninth) was selected. All teeth were coated with a 20 nm layer of gold and imaged on a Phillips XL 30 scanning electron microscope. All images were taken at 10 kV and a working distance of 88 mm. These images were used for morphological measurements. To provide a comparative estimate of tooth serrations, the total distance from the tip of the tooth to the base was measured along both the anterior and posterior edge. The distance along this edge showing serrations was recorded, and divided by the total distance to calculate the proportion of the tooth edge that was serrated (% serrations).

### 4.3. Proteomics Studies

#### 4.3.1. 1D Gel Electrophoresis

In order to establish the proteomics variation, 1D gradient gels were run under both reducing and non-reducing conditions using the manufacturer (BioRad) protocol. Gels were prepared as follows: 0.05 mL deionised H_2_O, 2.5 mL 30% acrylamide mix, 1.5 mL 1.0 M Tris-HCl, pH 8.45, 0.480 glycerol, 20 µL 10% APS, 2 µL TEMED (spreading gel); 0.760 mL deionised H_2_O, 0.760 mL 30% acrylamide mix, 0.760 mL 1.0 M Tris-HCl, pH 8.45, 15 µL 10% APS, 2 µL TEMED (spacer gel); 1.560 mL deionised H_2_O, 0.340 mL 30% acrylamide mix, 0.630 mL 1.0 M Tris-HCl, pH 8.45, 15 µL 10% APS, 2 µL TEMED (stacking gel). Spreading gel was cast first. After it was set the spacer gel was slowly layered atop of it, and after spacer gel was set the stacking gel was layered atop of it. Running buffers were: 0.2 M Tris-HCl, pH 8.9 (anode buffer); 0.1 M Tris-tricine-HCl pH 8.45. The gels were run at 100 V for three hours at room temperature. 30 µg of venom was reconstituted in Tricine loading buffer (Bio-Rad) with 10 mM DTT added to provide reduced conditions. Gels were stained overnight with colloidal Coomassie brilliant blue G250 (34% methanol, 3% phosphoric acid, 170 g/L ammonium sulphate, 1 g/L Coomassie blue G250). After the staining was complete, water was used to remove excess dye.

#### 4.3.2. 2D Gel Electrophoresis 

In order to further investigate the proteomics variation, particularly that of isoelectric variation, 2D gels were run using protocols previously optimised in the Fry lab [128,129,130]. 0.3 mg of venom sample were solubilized in 125 µL of rehydration buffer (8 M urea, 100 mM DTT, 4% CHAPS, and 0.5% ampholytes (Biolytes pH 3–10, Bio-Rad Lab)) with 0.01% bromophenol blue. The sample was mixed with shaking and centrifuged for 5 min at 4 °C, 14,000 rpm. This was done to remove any insoluble material. The supernatant was loaded onto IEF strips (Bio-Rad ReadyStrip, non-linear pH 3–10, 7 cm and 17 cm IPG) and left overnight for passive rehydration. Protein focussing was achieved via PROTEAN i12 IEF CELL (Bio-Rad Lab). The IEF running conditions were as follows: 100 V for 1 h, 500 V for 1 h, 1000 V for 1 h and 8000 V until 98,400 V/h. Actual current in the final step of the run varied in accordance to resistance. To each strip, a constant current of 50 µA was applied. After the run, IPG strips were incubated for 10 min in a reducing equilibration buffer (50 mM Tris–HCl, pH 8.8, 6 M urea, 2% SDS, 30% glycerol, 2% DTT) to reduce cysteine bonds. To alkylate reduced bonds, IPG strips were further incubated for 20 min in an alkylating equilibration buffer (50 mM Tris–HCl, pH 8.8, 6 M urea, 2% SDS, 30% glycerol, 2.5% iodoacetamide). After rinsing with SDS-PAGE running buffer, IPG strips were positioned on top of 12% polyacrylamide gels (Protean-II Plus, 18 × 20 cm, Bio-Rad Lab) using 0.5% agarose. Gels were run at 4 °C with a current of 10 mA per gel for 20 min followed by 20 mA per gel for the rest of the run until the bromophenol dye front was within 0.5 cm of the base of the gel. After the run, gels were briefly washed with water and stained with 0.2% colloidal Coomassie brilliant blue G250 overnight. Water was used to remove the excess of the dye after staining was complete. Visible spots were subsequently picked from gels and digested overnight at 37 °C with the use of sequencing grade trypsin (Sigma-Aldrich). Afterwards gel spots were washed with deionised H_2_O, destained (40 mM NH_4_CO_3_/50% acetonitrile (ACN)) and dehydrated (100% ACN), rehydrated in 10 µL of 20 µg/mL TPCK trypsin, and incubated at 37 °C overnight. To elute peptides, the following solutions were used per each gel spot: 20 µL of 1% formic acid (FA), followed by 20 µL of 5% ACN/0.5% FA. Collected peptides were put into MS vials and subjected to LC–MS/MS analysis.

#### 4.3.3. Shotgun Sequencing

In order to identify low molecular weight peptides that do not resolve well on 1D or 2D gels, shotgun sequencing was used. 3 µg of crude venom sample was dissolved in 50 µL of 100 mM ammonium carbonate to reduce and alkylate cysteine bonds with subsequent addition of 50 µL of 2% iodoethanol/0.5% triethylphosphine in acetonitrile. The sample was afterwards resuspended in 20 µL of 40 mM ammonium bicarbonate before overnight incubation (at 37 °C) with 750 ng of sequencing grade trypsin (Sigma-Aldrich). To stop digestion 1 µL of concentrated formic acid was added to each of the samples. Samples were lyophilised then resuspended in 20 µL of 5% ACN/0.5% FA, put into MS vials and subjected to LC–MS/MS analysis.

#### 4.3.4. LC–MS/MS

In order to identify the toxin types present, digested gel spots and digested whole venom (shotgun) samples were processed using an Agilent Zorbax stable bond C18 column (Agilent, Santa Clara, CA, USA) (2.1 mm by 100 mm, 1.8 µm particle size, 300 Å pore size) at a flow rate of 400 µL per minute and a gradient of 1–40% solvent B (90% acetonitrile, 0.1% formic acid) in 0.1% formic acid over 15 min or 4 min for shotgun samples and 2D-gel spots respectively on a Shimadzu Nexera UHPLC (Kyoto, Japan) coupled with an SCIEX 5600 Triple TOF mass spectrometer (Framingham, MA, USA). MS2 spectra are acquired at a rate of 20 scans per second with a cycle time of 2.3 s and optimised for high resolution. Precursor ions were selected between 80 and 1800 *m/z* with a charge state of 2–5 and of an intensity of at least 120 counts per second with a precursor selection window of 1.5 Da. The isotopes within 2 Da were excluded for MS2. MS2 spectra were searched against known translated transcriptome libraries or UniProt database with Proteinpilot v4.0 (Sciex Framingham, MA, USA) using a thorough identification search, specifying iodoacetamide as an alkylation method, trypsin digestion and allowing for biological and chemical modifications (ethanolyl C or deamidated N in particular) and amino acid substitutions, including artefacts induced by the preparation or analysis processes. This was done to maximize the identification of protein sequences. In order to remove false positives, only 95% confidence value hits were examined.

### 4.4. Bioactivity Studies

#### 4.4.1. Kallikrein Activity

##### RDES0011 Substrate

A working stock solution of freeze dried venom was reconstituted in a buffer containing 50% deionised H_2_O/50% glycerol (>99.9%, Sigma-Alrich) at a 1:1 ratio to preserve enzymatic activity and reduce enzyme degradation with the final venom concentration of 0.1 mg/mL, and then stored at −20 °C. Venom solutions (1 µg in dry venom weight) were plated in triplicates on a 384 well plate and measured by adding 90 µL quenched fluorescent substrate per well (total volume 100 µL/well; 10 µL/5mL enzyme buffer—150 mM NaCl and 50 mM Tri-HCl pH 7.4, Fluorogenic Peptide Substrate, R & D systems Cat#ES0011, Minneapolis, MN, USA). Fluorescence was monitored (excitation at 390 nm and emission at 460 nm) over 400 min or until activity ceased. 

##### S-2302 Substrate

Lyophilised venom sample reconstituted in PBS Ph7.4 (Lonza, Cat#17-516F) at 1 mg/mL. 0.5 ug of each venom sample was diluted in 100 μL of assay buffer—50 mM Tris, 50 mM NaCl, pH 8.8, 0.01% Tween 20 and plated in triplicate in a 96 well clear plastic plate. Chromogenic substrate S-2302 (H-D-Pro-Phe-Arg-pNA 2HCl) (Chromogenix, Partille, Sweden) reconstituted in 50% DMSO, 50% ddH_2_O at 16.4 mM. Substrate diluted to a 2× working solution in assay buffer at 200 µM, 100 µL of substrate solution added to each venom sample to a final substrate concentration of 100 µM. Absorbance monitored at 405 nm every 30 s for 100 min with FLUOstar Omega plate reader (BMG Labtech, Ortenberg, Germany).

#### 4.4.2. Phsopholipase A_2_ Activity

We assessed the continuous PLA_2_ activity of the venoms using a fluorescence substrate assay (EnzChek^®^ Phospholipase A_2_ Assay Kit) (ThermoFisher Scientific, Sydney, Australia). A working stock solution of freeze dried venom was reconstituted in a buffer containing 50% deionised H_2_O/50% glycerol (99.9%, Sigma) at a 1:1 ratio to preserve enzymatic activity and reduce enzyme degradation with the final venom concentration of 0.1 mg/mL, and then stored at −20 °C. Venom solution (0.1 µg in dry venom weight) was brought up to 12.5 µL in 1× PLA_2_ reaction buffer (250 mM Tris-HCL, 500 mM NaCl, 5 mM CaCl_2_, pH 8.9) and plated out in triplicates on a 384 well plate. Triplicates were measured by adding 12.5 µL quenched 1 mM EnzChek^®^ Phospholipase A_2_ substrate per well (total volume 25 µL/well) over 100 cycles at an excitation of 485 nm and emission of 520 nm, using a Fluoroskan Ascent (ThermoFisher Scientific). The negative control consisted of PLA_2_ reaction buffer and substrate only. 

#### 4.4.3. Rat ileum Organ Bath Testings

The rat ileum muscle preparations were isolated from adult male rats. The rats were euthanised by CO_2_ asphyxiation. The isolated preparations were individually mounted in 15 mL parallel organ baths containing a Krebs solution with the following constituents (mM): NaCl, 118.4; KCl, 4.7; MgSO_4_, 1.2; KH_2_PO_4_, 1.2; CaCl_2_, 2.5; NaHCO_3_, 25 and glucose, 11.1. The Krebs solution was continuously bubbled with carbogen (95% O_2_ and 5% CO_2_) to maintain a pH between 7.2–7.4 at a temperature of 32–34 °C. A resting tension between 1 and 3 g was found to be the optimal starting baseline. Stimulation was performed with 50 μg/mL of crude venom; deionised H_2_O (170 μL) was used as a control. The venom was left in the organ bath with the preparation for approximately 30 min or until the twitch response was completely abolished. Studies were conducted under University of Queensland Animal Ethics Committee approval # SBMS/310/12 (30 March 2012).

#### 4.4.4. Fibrinogen Gels

1 mm 12% SDS-PAGE gels were prepared using the following recipe for resolving gel layer: 3.3 mL deionised H_2_O, 2.5 mL 1.5 M Tris-HCl buffer pH 8.8 (Tris—Sigma-Aldrich, St. Louis, MO, USA; HCl—Univar, Wilnecote, UK), 100 μL 10% SDS (Sigma-Aldrich, St. Louis, MO, USA), 4 mL 30% acrylamide mix (Bio-Rad, Hercules, CA, USA), 100 μL 10% APS (Bio-Rad, Hercules, CA, USA), 4 μL TEMED (Bio-Rad, Hercules, CA, USA); and stacking gel layer: 1.4 mL deionised H_2_O, 250 μL 0.5 M Tris-HCl buffer pH 6.8, 20 μL 10% SDS (Sigma-Aldrich, St. Louis, MO, USA), 330 mL 30% acrylamide mix (Bio-Rad, Hercules, CA, USA), 20 μL 10% APS (Bio-Rad, Hercules, CA, USA), 2 μL TEMED (Bio-Rad, Hercules, CA, USA). 10× gel running buffer was prepared using the following recipe: 250 mM Tris (Sigma-Aldrich, St. Louis, MO, USA), 1.92 M glycine (MP Biomedicals, Santa Ana, CA, USA), 1% SDS (Sigma-Aldrich, St. Louis, MO, USA), pH 8.3.

Human fibrinogen was reconstituted to a concentration of 2 mg/mL in isotonic saline solution, flash frozen in liquid nitrogen and stored at −80 °C until use. Freeze-dried venom was reconstituted in deionised H_2_O and concentrations were measured using a Thermo Fisher Scientific™ NanoDrop 2000 (Waltham, MA, USA). Assay concentrations were a 1:10 ratio of venom:fibrinogen, in comparison to 1:5 ratios used in snake venom testing [131]). The following was conducted in triplicate for each venom: Five “secondary” aliquots containing 10 μL buffer (5 μL of 4× Laemmli sample buffer (Bio-Rad, Hercules, CA, USA), 5 μL deionised H_2_O, 100 mM DTT (Sigma-Aldrich, St. Louis, MO, USA)) were prepared. A “primary” aliquot of fibrinogen (volume/concentration as per the above) was warmed to 37 °C in an incubator. 10 μL was removed from the primary aliquot (“0 min incubation” fibrinogen control) and added to a secondary aliquot, pipette mixed, and boiled at 100 °C for 4 min. Assay concentrations were a 1:10 ratio of venom:fibrinogen, in comparison to 1:5 ratios used in snake venom testing [131]). The following was conducted in triplicate for each venom: Five “secondary” aliquots containing 10 μL buffer (5 μL of 4× Laemmli sample buffer (Bio-Rad, Hercules, CA, USA), 5 μL deionised H_2_O, 100 mM DTT (Sigma-Aldrich, St. Louis, MO, USA)) were prepared. A “primary” aliquot of fibrinogen (volume/concentration as per the above) was warmed to 37 °C in an incubator. 10 μL was removed from the primary aliquot (“0 min incubation” fibrinogen control) and added to a secondary aliquot, pipette mixed, and boiled at 100 °C for 4 min. 4 μg (dry weight) of venom was then added to the primary aliquot of fibrinogen (amounting to 0.1 mg/mL of venom and 1 mg/mL of fibrinogen in 40 μL total volume), pipette mixed, and immediately returned to the incubator. At each incubation time period (1 min, 5 min, 20 min, and 60 min), 10 μL was taken from the primary aliquot, added to a secondary aliquot, pipette mixed, and boiled at 100 °C for 4 min. At each incubation time period (1 min, 5 min, 20 min, and 60 min), 10 μL was taken from the primary aliquot, added to a secondary aliquot, pipette mixed, and boiled at 100 °C for 4 min. The secondary aliquots were then loaded into the gels and were run in 1× gel running buffer at room temperature for 20 min at 90 V (Mini Protean3 power-pack from Bio-Rad, Hercules, CA, USA) and then 120 V until the dye front neared the bottom of the gel. Gels were stained with colloidal coomassie brilliant blue G250 (34% methanol (VWR Chemicals, Tingalpa, QLD, Australia), 3% orthophosphoric acid (Merck, Darmstadt, Germany), 170 g/L ammonium sulfate (Bio-Rad, Hercules, CA, USA), 1 g/L coomassie blue G250 (Bio-Rad, Hercules, CA, USA), and destained in deionised H_2_O.

### 4.5. Bioinformatics

#### 4.5.1. Phylogenetic Comparative Analyses

A phylogeny was assembled using previous genetic studies [65,66,67] and was used for all further analyses conducted in R version 3.2.5 [132] using the ape package [133] for general handling of phylogenetic and trait data. Ancestral states were estimated and reconstructed over the tree in order to investigate the evolutionary history of the traits and consequently their relation to one another over time. The continuous functional traits were reconstructed by maximum likelihood in the contMap function in phytools [134]. We then fit PGLS models [135] in caper [136] to test for relationships. 

#### 4.5.2. Phylogenetic Reconstruction

Kallikrein, lysosomal acid lipase and chitinase datasets were analysed using Bayesian inference implemented on MrBayes, version 3.2.1 using lset rates = invgamma with prset aamodelpr = mixed, which enables the program to optimize between nine different amino acid substitution matrices. The analysis was performed by running a minimum of 10 million generations in four chains, and saving every 100th tree. The log-likelihood score of each saved tree was plotted against the number of generations to establish the point at which the log likelihood scores reached their asymptote, and the posterior probabilities for clades established by constructing a majority-rule consensus tree for all trees generated after completion of the burn-in phase.

#### 4.5.3. Molecular Modelling 

Publicly available kallikrein sequences were retrieved from GenBank by using *Varanus komodoensis* kallikrein sequence as a query for a BLAST search within Anguimorpha [137,138,139]. Sequences with less than 70% coverage were discarded. Sequences were edited to only include the codons for the mature protein using AliView and aligned by using AliView’s “Realign everything as translated amino acids” to translate the codons, align the resulting amino acids using MUSCLE, and reverse translating them [140,141]. MrBayes version 3.2 was used to create a phylogenetic tree of the sequences for performing the later selection analyses [142]. Using the closest sequence similarity reptile venom structure (GU441485) as input, the Phyre2 webserver generated a custom protein structure based on the published structure (PDB ID: 3S9C) [143]. This structure has a resolution of 1.8 Å and was 41% identical with the query sequence and with conservation of the cysteine residues. Protein models were rendered in UCSF Chimera version 1.10.2 [144]. Conservation scores were calculated using the UCSF Chimera implementation of AL2CO under the default settings [144,145]. Tests for selection were performed using HyPhy version 2.220150316: overall dN/dS value was calculated using the AnalyzeCodonData method, persistent site-by-site selection was analyzed with the FUBAR method, and episodic site-by-site selection was analyzed with the MEME method [146,147,148]. MEME is used for identifying sites that experience episodic selection pressures, where as FUBAR is improvement on site-wide selection analysis [148].

## Figures and Tables

**Table 1:** Toxins from anguimorph lizard venoms with characterised activities.

Toxin Type	Species Recovered from	Activity	References
AVIT	*V. komodoensis*, *V. varius*	While lizard forms have yet to be tested, snake venom homologues have been shown to have potent ability to contract smooth muscle and also induce hyperalgesia	[23,33,68]
BNP (B-type natriuretic peptide)	*C. warreni*, *G. infernalis*, *H. horridum*, *H. suspectum*, *V. glauerti*, *V. komodoensis*, *V. scalaris*, *V. varius*	Hypotension mediated by relaxation of aortic smooth muscle	[23,27,32,33,62,69]
Cholecystoxin	*V. varius*	Binding to cholecystokinin receptor to stimulate smooth muscle contraction	[27]
CRiSP (cysteine rich secretory protein)	*C. warreni*, *G. infernalis*, *H. horridum*, *H. suspectum*, *O. apodus*, *S. crocodilurus*, *V. acanthurus*, *V. albigularis*, *V. eremius*, *V. giganteus*, *V. gilleni*, *V. glauerti*, *V. gouldii*, *V. indicus*, *V. komodoensis*, *V. mertensi*, *V. mitchelli*, *V. scalaris*, *V. tristis*, *V. varius*	Blockage of ryanodine receptors and potassium channels, producing lethargy, paralysis, and hypothermia	[23,32,33,69,70,71,72,73,74]
Celestoxin	*C. warreni*	Hypotension inducing	[27]
Exendin	*H. exasperatu*, *H. horridum*, *H. suspectum*	Hypotension mediated by relaxation of aortic smooth muscle	[27,75,76,77]
Goannatyrotoxin	*V. eremius*	Hypertensive/hypotensive triphasic effect	[27]
Helofensin	*H. exasperatum*, *H. horridum*, *H. suspectum*	Blockage of nerve impulse	[27,78]
Helokinestatin	*C. warreni*, *G. infernalis*, *H. horridum*, *H. suspectum*	Bradykinin inhibition	[23,27,32,33,60,62]
Kallikrein	*C. warreni*, *G. infernalis*, *H. horridum*, *H. suspectum*, *O. apodus*, *V. gilleni*, *V. glauerti*, *V. scalaris*, *V. tristis*, *V. varius*	Kinin release from kininogen, cleavage of fibrinogen	[23,32,33,45,79,80,81,82,83,84,85]
Type III phospholipase A_2_ (PLA_2_)	*C. warren*, *H. horridum*, *H. suspectum*, *O. apodus*, *S. crododilurus*, *V. acanthurus*, *V. albigularis*, *V. eremius*, *V. giganteus*, *V. gilleni*, *V. glauerti*, *V. gouldii*, *V. indicus*, *V. komodoensis*, *V. mertensi*, *V. mitchelli*, *V. scalaris*, *V. tristis*, *V. varius*	Inhibition of epinephrine-induced platelet aggregation	[23,32,86]

**Table 2:** Toxin types recovered by proteomic analyses.

Species	AVIT	Chitinase	CRiSP	ESP	Kallikrein	Lysosomal Acid Lipase	Natriuretic Peptide	PLA_2_
*V. acanthurus*		+	+		+	+		
*V. baritji*		+	+		+	+		
*V. giganteus*			+	+	+	+	+	+
*V. gilleni*		+	+	+	+	+	+	+
*V. griseus*	+		+		+	+		+
*V. jobiensis*	+		+	+	+		+	+
*V. komodoensis*			+	+	+	+	+	+
*V. melinus*	+		+		+		+	+
*V. mertensi*			+	+	+	+	+	+
*V. mitchelli*			+	+	+	+	+	
*V. panoptes rubidus*			+	+	+			
*V. prasinus*			+		+	+		+
*V. salvadorii*	+		+		+	+	+	+
*V. scalaris*		+	+		+	+	+	
*V. tristis*		+	+		+	+	+	+
*V. varius*	+		+	+	+	+	+	+

**Table 3:** Normalised potency in bioactivity assays *.

Species	Substrate RDSE011	Substrate S-2302	Fibrinogen (Alpha-Chain)	Fibrinogen (Beta-Chain)	PLA_2_
*H. exasperatum*	0.300 ± 0.019	0.04 ± 0.003	0.435 ± 0.013	0.056 ± 0.002	0.200 ± 0.023
*H. horridum*	0.420 ± 0.027	0.408 ± 0.037	0.396 ± 0.015	0.159 ± 0.01	0.100 ± 0.009
*H. suspectum*	0.400 ± 0.015	0.103 ± 0.012	0.416 ± 0.015	0.103 ± 0.006	0.150 ± 0.017
*L. borneensis*	0.008 ± 0.001	0.102 ± 0.007	0.652 ± 0.018	0.458 ± 0.012	0.010 ± 0.007
*V. acanthurus*	0.059 ± 0.003	0.467 ± 0.022	0.818 ± 0.099	0.587 ± 0.066	0.050 ± 0.003
*V. baritji*	0.001 ± 0.001	0.874 ± 0.043	0.773 ± 0.047	0.308 ± 0.006	0.001 ± 0.001
*V. giganteus*	0.002 ± 0.001	0.351 ± 0.043	0.746 ± 0.034	0.538 ± 0.011	0.014 ± 0.009
*V. gilleni*	0.010 ± 0.003	0.400 ± 0.015	0.118 ± 0.002	0.001 ± 0.001	0.024 ± 0.003
*V. griseus*	0.006 ± 0.002	0.040 ± 0.009	0.247 ± 0.023	0.166 ± 0.003	0.015 ± 0.003
*V. jobiensis*	0.198 ± 0.013	0.317 ± 0.017	0.807 ± 0.026	0.882 ± 0.032	0.019 ± 0.002
*V. komodoensis*	0.080 ± 0.009	0.467 ± 0.012	0.339 ± 0.035	0.050 ± 0.001	0.011 ± 0.005
*V. melinus*	0.276 ± 0.015	0.317 ± 0.025	0.600 ± 0.016	0.503 ± 0.013	0.288 ± 0.019
*V. mertensi*	0.094 ± 0.004	0.300 ± 0.019	0.571 ± 0.018	0.546 ± 0.009	0.012 ± 0.005
*V. mitchelli*	1.000 ± 0.057	1.000 ± 0.097	0.962 ± 0.016	0.938 ± 0.053	0.008 ± 0.003
*V. panoptes rubidus*	0.208 ± 0.016	0.351 ± 0.011	0.230 ± 0.021	0.071 ± 0.003	0.019 ± 0.004
*V. prasinus*	0.407 ± 0.036	0.874 ± 0.057	0.700 ± 0.058	0.607 ± 0.027	0.028 ± 0.002
*V. salvadorii*	0.208 ± 0.012	0.103 ± 0.012	0.336 ± 0.015	0.225 ± 0.006	0.046 ± 0.007
*V. scalaris*	0.378 ± 0.019	1.000 ± 0.089	1.000 ± 0.037	1.000 ± 0.057	0.119 ± 0.011
*V. tristis*	0.161 ± 0.015	0.040 ± 0.003	0.793 ± 0.021	0.361 ± 0.009	0.017 ± 0.005
*V. varius*	0.072 ± 0.009	0.226 ± 0.013	0.377 ± 0.023	0.286 ± 0.011	1.000 ± 0.022

* See Supplementary Table S1 for raw values.

## Supplementary Materials

The following are available online at www.mdpi.com/2072-6651/9/8/242/s1. Table S1: MSMS data, Table S2 and Table S3 tests for significance.xlsx.

## References

[1] Auffenberg W. (1981). Behavioral Ecology of the Komodo Monitor.

[2] Montgomery J.M., Gillespie D., Sastrawan P., Fredeking T.M., Stewart G.L. (2002). Aerobic salivary bacteria in wild and captive Komodo dragons. J. Wildl. Dis..

[3] Goldstein E.J., Tyrrell K.L., Citron D.M., Cox C.R., Recchio I.M., Okimoto B., Bryja J., Fry B.G. (2013). Anaerobic and aerobic bacteriology of the saliva and gingiva from 16 captive Komodo dragons (*Varanus komodoensis*): New implications for the “bacteria as venom” model. J. Zoo Wildl. Med..

[4] Hocknull S.A., Piper P.J., van den Bergh G.D., Due R.A., Morwood M.J., Kurniawan I. (2009). Dragon’s paradise lost: Palaeobiogeography, evolution and extinction of the largest-ever terrestrial lizards (Varanidae). PLoS ONE.

[5] Fry B.G., Scheib H., Messenger K., Hocknull S., Wroe S., Sunagar K., Goldstein E.J.C., Tyrrell K.L., Citron D.M., Jackson T.N.W., Fry B.G. (2015). Poisonous snakes and bacteria as a Komodo dragon weapon: Which is a myth and which is reality?. Venomous Reptiles: Evolution, Pathophysiology and Biodiscovery.

[6] Estes R., Estes R., Pregill G. (1988). Charles L. Camp—An appreciation. Phylogenetic Relationships of the Lizard Families: Essay Commemorating Charles L. Camp.

[7] Losos J.B., Hillis D.M., Greene H.W. (2012). Evolution. Who speaks with a forked tongue?. Science.

[8] Sweet S.S., Pianka E.R. (2007). Monitors, mammals and Wallace’s line. Mertensiella.

[9] Vitt L.J. (2013). Walking the Natural-History Trail. Herpetologica.

[10] Sweet S.S., Conta M. (2016). Chasing Flamingos: Toxicofera and the Misinterpretation of Venom in Varanid Lizards. Proceedings of the 2015 Interdisciplinary World Conference on Monitor Lizards.

[11] Hedges S., Vidal N., Hedges S.B., Kumar S. (2009). Lizards, snakes, and amphisbaenians (Squamata). The Timetree of Life.

[12] Pyron R.A., Burbrink F.T. (2012). Extinction, ecological opportunity, and the origins of global snake diversity. Evolution.

[13] Pyron R.A., Burbrink F.T., Wiens J.J. (2013). A phylogeny and revised classification of Squamata, including 4161 species of lizards and snakes. BMC Evol. Biol..

[14] Reeder T.W., Townsend T.M., Mulcahy D.G., Noonan B.P., Wood P.L., Sites J.W., Wiens J.J. (2015). Integrated analyses resolve conflicts over squamate reptile phylogeny and reveal unexpected placements for fossil taxa. PLoS ONE.

[15] Townsend T., Larson A., Louis E., Macey J.R. (2004). Molecular phylogenetics of squamata: The position of snakes, amphisbaenians, and dibamids, and the root of the squamate tree. Syst. Biol..

[16] Vidal N., David P. (2004). New insights into the early history of snakes inferred from two nuclear genes. Mol. Phylogenet. Evol..

[17] Vidal N., Hedges S.B. (2005). The phylogeny of squamate reptiles (lizards, snakes, and amphisbaenians) inferred from nine nuclear protein-coding genes. C. R. Biol..

[18] Vidal N., Hedges S.B. (2009). The molecular evolutionary tree of lizards, snakes, and amphisbaenians. C. R. Biol..

[19] Wiens J.J., Hutter C.R., Mulcahy D.G., Noonan B.P., Townsend T.M., Sites J.W., Reeder T.W. (2012). Resolving the phylogeny of lizards and snakes (Squamata) with extensive sampling of genes and species. Biol. Lett..

[20] Wiens J.J., Kuczynski C.A., Townsend T., Reeder T.W., Mulcahy D.G., Sites J.W. (2010). Combining Phylogenomics and Fossils in Higher-Level Squamate Reptile Phylogeny: Molecular Data Change the Placement of Fossil Taxa. Syst. Biol..

[21] Pyron R.A. (2017). Novel Approaches for Phylogenetic Inference from Morphological Data and Total-Evidence Dating in Squamate Reptiles (Lizards, Snakes, and Amphisbaenians). Syst. Biol..

[22] Zheng Y., Wiens J.J. (2016). Combining phylogenomic and supermatrix approaches, and a time-calibrated phylogeny for squamate reptiles (lizards and snakes) based on 52 genes and 4162 species. Mol. Phylogenet. Evol..

[23] Fry B.G., Vidal N., Norman J.A., Vonk F.J., Scheib H., Ramjan S.F., Kuruppu S., Fung K., Hedges S.B., Richardson M.K. (2006). Early evolution of the venom system in lizards and snakes. Nature.

[24] Fry B.G. (2005). From genome to “venome”: Molecular origin and evolution of the snake venom proteome inferred from phylogenetic analysis of toxin sequences and related body proteins. Genome Res..

[25] Fry B.G., Casewell N.R., Wuster W., Vidal N., Young B., Jackson T.N. (2012). The structural and functional diversification of the Toxicofera reptile venom system. Toxicon.

[26] Fry B.G., Roelants K., Champagne D.E., Scheib H., Tyndall J.D., King G.F., Nevalainen T.J., Norman J.A., Lewis R.J., Norton R.S. (2009). The toxicogenomic multiverse: Convergent recruitment of proteins into animal venoms. Annu. Rev.Genom. Hum. Genet..

[27] Fry B.G., Roelants K., Winter K., Hodgson W.C., Griesman L., Kwok H.F., Scanlon D., Karas J., Shaw C., Wong L. (2010). Novel venom proteins produced by differential domain-expression strategies in beaded lizards and gila monsters (genus *Heloderma*). Mol. Biol. Evol..

[28] Fry B.G., Scheib H., van der Weerd L., Young B., McNaughtan J., Ramjan S.F., Vidal N., Poelmann R.E., Norman J.A. (2008). Evolution of an arsenal: Structural and functional diversification of the venom system in the advanced snakes (Caenophidia). Mol. Cell. Proteom..

[29] Fry B.G., Sunagar K., Casewell N.R., Kochva E., Roelants K., Scheib H., Wüster W., Vidal N., Young B., Burbrink F., Fry B.G. (2015). The origin and evolution of the Toxicofera reptile venom system. Venomous Reptiles and Their Toxins: Evolution, Pathophysiology and Biodiscovery.

[30] Fry B.G., Undheim E.A., Ali S.A., Jackson T.N., Debono J., Scheib H., Ruder T., Morgenstern D., Cadwallader L., Whitehead D. (2013). Squeezers and leaf-cutters: Differential diversification and degeneration of the venom system in toxicoferan reptiles. Mol. Cell. Proteom..

[31] Fry B.G., Vidal N., van der Weerd L., Kochva E., Renjifo C. (2009). Evolution and diversification of the Toxicofera reptile venom system. J. Proteom..

[32] Fry B.G., Winter K., Norman J.A., Roelants K., Nabuurs R.J., van Osch M.J., Teeuwisse W.M., van der Weerd L., McNaughtan J.E., Kwok H.F. (2010). Functional and structural diversification of the Anguimorpha lizard venom system. Mol. Cell. Proteom..

[33] Fry B.G., Wroe S., Teeuwisse W., van Osch M.J., Moreno K., Ingle J., McHenry C., Ferrara T., Clausen P., Scheib H. (2009). A central role for venom in predation by *Varanus komodoensis* (Komodo Dragon) and the extinct giant *Varanus* (*Megalania*) *priscus*. Proc. Natl. Acad. Sci. USA.

[34] Fry B.G., Wuster W. (2004). Assembling an arsenal: Origin and evolution of the snake venom proteome inferred from phylogenetic analysis of toxin sequences. Mol. Biol. Evol..

[35] Mackessy S.P., Saviola A.J. (2016). Understanding biological roles of venoms among the caenophidia: The importance of rear-fanged snakes. Integr. Comp. Biol..

[36] Hsiang A.Y., Field D.J., Webster T.H., Behlke A.D., Davis M.B., Racicot R.A., Gauthier J.A. (2015). The origin of snakes: Revealing the ecology, behavior, and evolutionary history of early snakes using genomics, phenomics, and the fossil record. BMC Evol. Biol..

[37] Sites J.W., Reeder T.W., Wiens J.J. (2011). Phylogenetic insights on evolutionarynovelties in lizards and snakes: Sex, birth, bodies, niches, and venom. Annu. Rev. Ecol. Evolut. Syst..

[38] Weinstein S.A. (2015). Snake venoms: A brief treatise on etymology, origins of terminology, and definitions. Toxicon.

[39] Jackson T.N.W., Young B., McCarthy C.J., Kochva E., Vidal N., Underwood G., Fry B.G. (2017). Endless forms most beautiful: The evolution of ophidian oral glands, including the venom system, and the use of appropriate terminology for homologous structures. Zoomorphology.

[40] Hargreaves A.D., Swain M.T., Logan D.W., Mulley J.F. (2014). Testing the Toxicofera: Comparative transcriptomics casts doubt on the single, early evolution of the reptile venom system. Toxicon.

[41] Jackson T.N., Fry B.G. (2016). A Tricky Trait: Applying the Fruits of the “Function Debate” in the Philosophy of Biology to the “Venom Debate” in the Science of Toxinology. Toxins.

[42] Mebs D. (2002). Venomous and Poisonous Animals: A Handbook for Biologists, Toxicologists and Toxinologists, Physicians and Pharmacists.

[43] Hargreaves A.D., Tucker A.S., Mulley J.F., Malhotra A. (2017). A Critique of the Toxicoferan Hypothesis. Evolution of Venomous Animals and Their Toxins.

[44] Fry B.G., Wickramaratana J.C., Lemme S., Beuve A., Garbers D., Hodgson W.C., Alewood P. (2005). Novel natriuretic peptides from the venom of the inland taipan (*Oxyuranus microlepidotus*): Isolation, chemical and biological characterisation. Biochem. Biophys. Res. Commun..

[45] Mebs D. (1969). Purification and properties of a kinin liberating enzyme from venom of *Heloderma*
*suspectum*. Naunyn-Schmiedeberg’s Arch. Pharmakol..

[46] Gorelov Y.K. (1971). Concerning the *Varanus griseus* saliva toxicity. Izy. Akademii Turkmenistan SSR.

[47] Sopyev O., Makeev V.M., Kudryavtsev S.V., Makarov A.N. (1987). Case of intoxification from a bite of Varanus griseus. Izy. Akademii Turkmenistan SSR.

[48] Ballard V., Antonio F.B. (2001). *Varanus griseus* (Desert monitor) toxicity. Herpetol. Rev..

[49] Vikrant S., Verma B.S. (2014). Monitor lizard bite-induced acute kidney injury—A case report. Ren. Fail..

[50] White J., Weinstein S.A. (2015). Reply to Vikrant and Verma about “Monitor Lizard Envenoming”. Ren. Fail..

[51] Ducey S.D., Cooper J.S., Wadman M.C. (2016). Bitten by a Dragon. Wilderness Environ. Med..

[52] Loop M.S. (1974). The effect of relative prey size on the ingestion behavior of the Bengal monitor, *Varanus bengalensis* (Sauria: Varanidae). Herpetologica.

[53] Kochva E. (1978). Oral glands of the Reptilia. Physiology B.

[54] Li M., Fry B.G., Kini R.M. (2005). Eggs-Only diet: Its implications for the toxin profile changes and ecology of the marbled sea snake (*Aipysurus eydouxii*). J. Mol. Evol..

[55] Li M., Fry B.G., Kini R.M. (2005). Putting the brakes on snake venom evolution: The unique molecular evolutionary patterns of *Aipysuras eydouxii* (Marbled sea snake) phospholipase A(2) toxins. Mol. Biol. Evol..

[56] Morgenstern D., King G.F. (2013). The venom optimization hypothesis revisited. Toxicon.

[57] Koludarov I., Jackson T.N., Sunagar K., Nouwens A., Hendrikx I., Fry B.G. (2014). Fossilized venom: The unusually conserved venom profiles of *Heloderma* species (beaded lizards and gila monsters). Toxins.

[58] Furman B.L. (2012). The development of Byetta (exenatide) from the venom of the Gila monster as an anti-diabetic agent. Toxicon.

[59] Irwin D.M. (2012). Origin and convergent evolution of exendin genes. Gen. Comp. Endocrinol..

[60] Kwok H.F., Chen T., O’Rourke M., Ivanyi C., Hirst D., Shaw C. (2008). Helokinestatin: A new bradykinin B-2 receptor antagonist decapeptide from lizard venom. Peptides.

[61] Sanggaard K.W., Dyrlund T.F., Thomsen L.R., Nielsen T.A., Brøndum L., Wang T., Thøgersen I.B., Enghild J.J. (2015). Characterization of the gila monster (*Heloderma suspectum suspectum*) venom proteome. J. Proteom..

[62] Ma C., Yang M., Zhou M., Wu Y., Wang L., Chen T., Ding A., Shaw C. (2011). The natriuretic peptide/helokinestatin precursor from Mexican beaded lizard (*Heloderma horridum*) venom: Amino acid sequence deduced from cloned cDNA and identification of two novel encoded helokinestatins. Peptides.

[63] Ma C., Wang H., Wu Y., Zhou M., Lowe G., Wang L., Zhang Y., Chen T., Shaw C. (2012). Helokinestatin-7 peptides from the venoms of *Heloderma* lizards. Peptides.

[64] Zhang Y., Wang L., Zhou M., Zhou Z., Chen X., Chen T., Kwok H., Ivanyi C., Shaw C. (2010). The structure of helokinestatin-5 and its biosynthetic precursor from Gila monster (*Heloderma suspectum*) venom: Evidence for helokinestatin antagonism of bradykinin-induced relaxation of rat tail artery smooth muscle. Peptides.

[65] Ast J.C. (2001). Mitochondrial DNA evidence and evolution in Varanoidea (Squamata). Cladistics.

[66] Thompson G.G., Clemente C.J., Withers P.C., Fry B.G., Norman J.A. (2008). Is body shape of varanid lizards linked with retreat choice?. Aust. J. Zool..

[67] Vidal N., Marin J., Sassi J., Battistuzzi F.U., Donnellan S., Fitch A.J., Fry B.G., Vonk F.J., Rodriguez de la Vega R.C., Couloux A. (2012). Molecular evidence for an Asian origin of monitor lizards followed by Tertiary dispersals to Africa and Australasia. Biol. Lett..

[68] Schweitz H., Pacaud P., Diochot S., Moinier D., Lazdunski M. (1999). MIT1, a black mamba toxin with a new and highly potent activity on intestinal contraction. FEBS Lett..

[69] Fry B.G., Sunagar K., Jackson T.N.W., Reeks T., Kwok H.F., Fry B.G. (2015). B-type natriuretic peptides. Venomous Reptiles and Their Toxins: Evolution, Pathophysiology and Biodiscovery.

[70] Mochca-Morales J., Martin B.M., Possani L.D. (1990). Isolation and characterization of helothermine, a novel toxin from *Heloderma horridum horridum* (Mexican beaded lizard) venom. Toxicon.

[71] Morrissette J., Elhayek R., Possani L., Coronado R. (1994). Isolation and characterization of ryanodine receptor toxins from *Heloderma horridum* (mexican beaded lizard) venom. Biophys. J..

[72] Morrissette J., Krätzschmar J., Haendler B., El-Hayek R., Mochca-Morales J., Martin B.M., Patel J.R., Moss R.L., Schleuning W.-D., Coronado R. (1995). Primary structure and properties of helothermine, a peptide toxin that blocks ryanodine receptors. Biophys. J..

[73] Nobile M., Magnelli V., Lagostena L., Mochca-Morales J., Possani L.D., Prestipino G. (1994). The toxin helothermine affects potassium currents in newborn rat cerebellar granule cells. J. Membr. Biol..

[74] Nobile M., Noceti F., Prestipino G., Possani L.D. (1996). Helothermine, a lizard venom toxin, inhibits calcium current in cerebellar granules. Exp. Brain Res..

[75] Grundemar L., Högestätt E.D. (1990). Vascular effects of helodermin, helospectin I and helospectin II: A comparison with vasoactive intestinal peptide (VIP). Br. J. Pharmacol..

[76] Tsueshita T., Onyukusel H., Sethi V., Gandhi S., Rubinstein I. (2004). Helospectin I and II evoke vasodilation in the intact peripheral microcirculation. Peptides.

[77] Uddman R., Goadsby P.J., Jansen-Olesen I., Edvinsson L. (1999). Helospectin-like peptides: Immunochemical localization and effects on isolated cerebral arteries and on local cerebral blood flow in the cat. J. Cereb. Blood Flow Metabol..

[78] Komori Y., Nikai T., Sugihara H. (1988). Purification and characterization of a lethal toxin from the venom of *Heloderma horridum horridum*. Biochem. Biophys. Res. Commun..

[79] Datta G., Tu A.T. (1997). Structure and other chemical characterizations of gila toxin, a lethal toxin from lizard venom. J. Pept. Res..

[80] Mebs D. (1969). Isolation and properties of kallikrein from venom of gila monster (*Heloderma suspectum*). Hoppe-Seylers Z. Physiol. Chem..

[81] Nikai T., Imai K., Komori Y., Sugihara H. (1992). Isolation and characterization of arginine ester hydrolase from *Heloderma horridum* (beaded lizard) venom. Int. J. Biochem..

[82] Nikai T., Imai K., Nagasaka M., Sugihara H. (1988). Kallikrein-like enzyme from the venom of *Agkistrodon p. piscivorus*. Int. J. Biochem..

[83] Nikai T., Imai K., Sugihara H., Tu A.T. (1988). Isolation and characterization of horridum toxin with arginine ester hydrolase activity from *Heloderma horridum* (beaded lizard) venom. Arch. Biochem. Biophys..

[84] Utaisincharoen P., Mackessy S.P., Miller R.A., Tu A.T. (1993). Complete primary structure and biochemical properties of gilatoxin, a serine protease with kallikrein-like and angiotensin-degrading activities. J. Biol. Chem..

[85] Vaiyapuri S., Sunagar K., Gibbins J.M., Jackson T.N.W., Reeks T., Fry B.G., Fry B.G. (2015). Kallikrein Enzymes In Venomous Reptiles and Their Toxins: Evolution, Pathophysiology and Biodiscovery.

[86] Huang T.F., Chiang H.S. (1994). Effect on human platelet-aggregation of phospholipase a(2) purified from *Heloderma horridum* (beaded lizard) venom. Biochim. Biophys. Acta Lipids Lipid Metab..

[87] Salemi M., Vandamme A.-M. (2003). The Phylogenetic Handbook: A Practical Approach to DNA and Protein Phylogeny.

[88] Aminetzach Y.T., Srouji J.R., Kong C.Y., Hoekstra H.E. (2009). Convergent evolution of novel protein function in shrew and lizard venom. Curr. Biol..

[89] Boutemy L.S., King S.R.F., Win J., Hughes R.K., Clarke T.A., Blumenschein T.M.A., Kamoun S., Banfield M.J. (2011). Structures of Phytophthora RXLR Effector Proteins a conserved but adaptable fold underpins functional diversity. J. Biol. Chem..

[90] Brodie E.D. (2010). Convergent Evolution: Pick Your Poison Carefully. Curr. Biol..

[91] Davies K.T.J., Cotton J.A., Kirwan J.D., Teeling E.C., Rossiter S.J. (2012). Parallel signatures of sequence evolution among hearing genes in echolocating mammals: An emerging model of genetic convergence. Heredity.

[92] Folinsbee K.E. (2013). Evolution of venom across extant and extinct eulipotyphlans. C. R. Palevol.

[93] Garb J.E., Hayashi C.Y. (2013). Molecular evolution of alpha-latrotoxin, the exceptionally potent vertebrate neurotoxin in black widow spider venom. Mol. Biol. Evol..

[94] Green D.A., Extavour C.G. (2012). Convergent evolution of a reproductive trait through distinct developmental mechanisms in Drosophila. Dev. Biol..

[95] Guo S.H., Skala W.G., Magdolen V., Briza P., Biniossek M.L., Schilling O., Kellermann J., Brandstetter H., Goettig P. (2016). A Single Glycan at the 99-Loop of Human Kallikrein-related Peptidase 2 Regulates Activation and Enzymatic Activity. J. Biol. Chem..

[96] Harrington M. (2013). Exploring the molecular underpinnings of convergent evolution. Lab. Anim..

[97] Janes D.E., Organ C.L., Fujita M.K., Shedlock A.M., Edwards S.V., Chakravarti A., Green E. (2010). Genome Evolution in Reptilia, the Sister Group of Mammals. Annual Review of Genomics and Human Genetics.

[98] Lawrence M.G., Lai J., Clements J.A. (2010). Kallikreins on Steroids: Structure, Function, and Hormonal Regulation of Prostate-Specific Antigen and the Extended Kallikrein Locus. Endocr. Rev..

[99] Ligabue-Braun R., Verli H., Carlini C.R. (2012). Venomous mammals: A review. Toxicon.

[100] Losos J.B. (2011). Convergence, adaptation, and constraint. Evolution.

[101] Martin A., Orgogozo V. (2013). The loci of repeated evolution: A catalog of genetic hotspots of phenotypic variation. Evolution.

[102] Meyer W.K., Zhang S., Hayakawa S., Imai H., Przeworski M. (2013). The convergent evolution of blue iris pigmentation in primates took distinct molecular paths. Am. J. Phys. Anthropol..

[103] Pavlopoulou A., Pampalakis G., Michalopoulos I., Sotiropoulou G. (2010). Evolutionary History of Tissue Kallikreins. PLoS ONE.

[104] Roelants K., Fry B.G., Norman J.A., Clynen E., Schoofs L., Bossuyt F. (2010). Identical Skin Toxins by Convergent Molecular Adaptation in Frogs. Curr. Biol..

[105] Simmer J.P., Richardson A.S., Smith C.E., Hu Y.Y., Hu J.C.C. (2011). Expression of kallikrein-related peptidase 4 in dental and non-dental tissues. Eur. J. Oral Sci..

[106] Song B.X., Wang F., Guo Y., Sang Q., Liu M., Li D.Y., Fang W., Zhang D.L. (2012). Protein-protein interaction network-based detection of functionally similar proteins within species. Proteins-Struct. Funct. Bioinform..

[107] Von Reumont B.M., Blanke A., Richter S., Alvarez F., Bleidorn C., Jenner R.A. (2014). The first venomous crustacean revealed by transcriptomics and functional morphology: Remipede venom glands express a unique toxin cocktail dominated by enzymes and a neurotoxin. Mol. Biol. Evol..

[108] Wong E.S.W., Papenfuss A.T., Whittington C.M., Warren W.C., Belov K. (2012). A limited role for gene duplications in the evolution of platypus venom. Mol. Biol. Evol..

[109] Yennamalli R.M., Rader A.J., Wolt J.D., Sen T.Z. (2011). Thermostability in endoglucanases is fold-specific. BMC Struct. Biol..

[110] Zhu L.M., Peigneur S., Gao B., Zhang S.F., Tytgat J., Zhu S.Y. (2016). Target-driven positive selection at hot spots of scorpion toxins uncovers their potential in design of insecticides. Mol. Biol. Evol..

[111] Zhu S.Y., Gao B., Deng M.C., Yuan Y.Z., Luo L., Peigneur S., Xiao Y.C., Liang S.P., Tytgat J. (2010). Drosotoxin, a selective inhibitor of tetrodotoxin-resistant sodium channels. Biochem. Pharmacol..

[112] Zhu S.Y., Peigneur S., Gao B., Lu X.X., Cao C.Y., Tytgat J. (2012). Evolutionary diversification of mesobuthus alpha-scorpion toxins affecting sodium channels. Mol. Cell. Proteom..

[113] Schweitz H., Bidard J.N., Lazdunski M. (1990). Purification and pharmacological characterization of peptide toxins from the black mamba (*Dendroaspis polylepis*) venom. Toxicon.

[114] Mertens R. (1942). Die familie der warane (Varanidae). Abh. Senckenberg. Naturforschenden Ges..

[115] Rieppel O. (1979). A functional interpretation of the varanid dentition (Reptilia, Lacertilia, Varanidae). Gegenbaurs Morphol. Jahrb..

[116] Abler W.L. (1992). The serrated teeth of tyrannosaurid dinosaurs, and biting structures in other animals. Paleobiology.

[117] Rieppel O., Labhardt L. (1979). Mandibular mechanics in *Varanus niloticus* (Reptilia: Lacertilia). Herpetologica.

[118] Sweet S.S., Horn H.G., Boehme W., Krebs U. (2007). Comparative spatial ecology of two small arboreal monitors in northern Australia. Advances in Monitor Research III.

[119] Sunagar K., Moran Y. (2015). The rise and fall of an evolutionary innovation: Contrasting strategies of venom evolution in ancient and young animals. PLoS Genet..

[120] Pascoe J.H., Mulley R.C., Spencer R., Chapple R. (2012). Diet analysis of mammals, raptors and reptiles in a complex predator assemblage in the Blue Mountains, eastern Australia. Aust. J. Zool..

[121] Pianka E.R., King D., King R.A. (2004). Varanoid Lizards of the World.

[122] Bull J.J., Jessop T.S., Whiteley M. (2010). Deathly drool: Evolutionary and ecological basis of septic bacteria in Komodo dragon mouths. PLoS ONE.

[123] Arbuckle K. (2009). Ecological function of venom in *Varanus*, with a compilation of dietary records from the literature. Biawak.

[124] Grant T.R., Temple-Smith P.D. (1998). Field biology of the platypus (*Ornithorhynchus anatinus*): Historical and current perspectives. Philos. Trans. R. Soc. B Biol. Sci..

[125] Arbuckle K., Malhotra A., Gopalakrishnakone P. (2015). Evolutionary context of venom in animals. Evolution of Venomous Animals and Their Toxins.

[126] Harris R.J., Arbuckle K. (2016). Tempo and mode of the evolution of venom and poison in tetrapods. Toxins.

[127] Marsh N., Williams V. (2005). Practical applications of snake venom toxins in haemostasis. Toxicon.

[128] Ali S.A., Baumann K., Jackson T.N., Wood K., Mason S., Undheim E.A., Nouwens A., Koludarov I., Hendrikx I., Jones A. (2013). Proteomic comparison of *Hypnale hypnale* (Hump-Nosed Pit-Viper) and *Calloselasma rhodostoma* (Malayan Pit-Viper) venoms. J. Proteom..

[129] Ali S.A., Jackson T.N., Casewell N.R., Low D.H., Rossi S., Baumann K., Fathinia B., Visser J., Nouwens A., Hendrikx I. (2015). Extreme venom variation in Middle Eastern vipers: A proteomics comparison of *Eristicophis macmahonii*, *Pseudocerastes fieldi* and *Pseudocerastes persicus*. J. Proteom..

[130] Low D.H., Sunagar K., Undheim E.A., Ali S.A., Alagon A.C., Ruder T., Jackson T.N., Pineda Gonzalez S., King G.F., Jones A. (2013). Dracula’s children: Molecular evolution of vampire bat venom. J. Proteom..

[131] Weldon C.L., Mackessy S.P. (2010). Biological and proteomic analysis of venom from the Puerto Rican Racer (*Alsophis portoricensis*: Dipsadidae). Toxicon.

[132] R-Core-Team R: A Language and Environment for Statistical Computing. https://www.R-project.org/.

[133] Paradis E., Claude J., Strimmer K. (2004). APE: Analyses of phylogenetics and evolution in R language. Bioinformatics.

[134] Revell L.J. (2012). phytools: An R package for phylogenetic comparative biology (and other things). Methods Ecol. Evol..

[135] Symonds M.R.E., Blomberg S.P., Garamszegi L.Z. (2014). A Primer on Phylogenetic Generalised Least Squares. Modern Phylogenetic Comparative Methods and Their Application in Evolutionary Biology.

[136] Orme D., Freckleton R., Thomas G., Petzoldt T., Fritz S., Isaac N., Pearse W. (2013). The Caper Package: Comparative Analyses of Phylogenetics and Evolution in R, Version 0.5.2. https://cran.r-project.org/web/packages/caper/vignettes/caper.pdf.

[137] Altschul S.F., Gish W., Miller W., Myers E.W., Lipman D.J. (1990). Basic local alignment search tool. J. Mol. Biol..

[138] Benson D.A., Cavanaugh M., Clark K., Karsch-Mizrachi I., Lipman D.J., Ostell J., Sayers E.W. (2013). GenBank. Nucleic Acids Res..

[139] Benson D.A., Clark K., Karsch-Mizrachi I., Lipman D.J., Ostell J., Sayers E.W. (2014). GenBank. Nucleic Acids Res..

[140] Edgar R.C. (2004). MUSCLE: A multiple sequence alignment method with reduced time and space complexity. BMC Bioinform..

[141] Larsson A. (2014). AliView: A fast and lightweight alignment viewer and editor for large datasets. Bioinformatics.

[142] Ronquist F., Teslenko M., van der Mark P., Ayres D.L., Darling A., Hohna S., Larget B., Liu L., Suchard M.A., Huelsenbeck J.P. (2012). MrBayes 3.2: Efficient Bayesian phylogenetic inference and model choice across a large model space. Syst. Biol..

[143] Kelley L.A., Mezulis S., Yates C.M., Wass M.N., Sternberg M.J. (2015). The Phyre2 web portal for protein modeling, prediction and analysis. Nat. Protoc..

[144] Pettersen E.F., Goddard T.D., Huang C.C., Couch G.S., Greenblatt D.M., Meng E.C., Ferrin T.E. (2004). UCSF Chimera—A visualization system for exploratory research and analysis. J. Comput. Chem..

[145] Pei J., Grishin N.V. (2001). AL2CO: Calculation of positional conservation in a protein sequence alignment. Bioinformatics.

[146] Murrell B., Moola S., Mabona A., Weighill T., Sheward D., Kosakovsky Pond S.L., Scheffler K. (2013). FUBAR: A fast, unconstrained bayesian approximation for inferring selection. Mol. Biol. Evol..

[147] Murrell B., Wertheim J.O., Moola S., Weighill T., Scheffler K., Kosakovsky Pond S.L. (2012). Detecting individual sites subject to episodic diversifying selection. PLoS Genet..

[148] Pond S.L.K., Frost S.D.W., Muse S.V. (2005). HyPhy: Hypothesis testing using phylogenies. Bioinformatics.

