# Supplementary material for: Enter the Dragon: The Dynamic and Multifunctional Evolution of Anguimorpha Lizard Venoms
Source: Toxins (Basel). 2017 Aug 6;9(8):242. doi: 10.3390/toxins9080242 (PMC5577576; doi:10.3390/toxins9080242)
Supplement: Supplementary file 1 [file toxins-09-00242-s001.zip › toxins-204371 supplementary/toxins-204371 Supplementary Table 2 raw activity values.docx]

**Supplementary Table 1:** Raw data values

| Species | Substrate RDSE011 | Substrate S-2302 | Fibrinogen  (alpha-chain) | Fibrinogen  (beta-chain) | PLA_2_ |
| --- | --- | --- | --- | --- | --- |
| *H. exasperatum* | 3.394+/-0.215 | 0.021+/-0.002 | 1.69+/-0.051 | 0.139+/-0.005 | 0.51+/-0.059 |
| *H. horridum* | 4.752+/-0.305 | 0.212+/-0.019 | 1.538+/-0.058 | 0.395+/-0.025 | 0.255+/-0.023 |
| *H. suspectum* | 4.525+/-0.17 | 0.054+/-0.006 | 1.616+/-0.058 | 0.256+/-0.015 | 0.382+/-0.043 |
| *L. borneensis* | 0.091+/-0.011 | 0.053+/-0.004 | 2.533+/-0.07 | 1.139+/-0.03 | 0.025+/-0.018 |
| *V. acanthurus* | 0.667+/-0.034 | 0.243+/-0.011 | 3.178+/-0.385 | 1.46+/-0.164 | 0.127+/-0.008 |
| *V. baritji* | 0.011+/-0.011 | 0.454+/-0.022 | 3.003+/-0.183 | 0.766+/-0.015 | 0.003+/-0.003 |
| *V. giganteus* | 0.023+/-0.011 | 0.183+/-0.022 | 2.898+/-0.132 | 1.338+/-0.027 | 0.036+/-0.023 |
| *V. gilleni* | 0.113+/-0.034 | 0.208+/-0.008 | 0.458+/-0.008 | 0.002+/-0.002 | 0.061+/-0.008 |
| *V. griseus* | 0.068+/-0.023 | 0.021+/-0.005 | 0.96+/-0.089 | 0.413+/-0.007 | 0.038+/-0.008 |
| *V. jobiensis* | 2.24+/-0.147 | 0.165+/-0.009 | 3.135+/-0.101 | 2.193+/-0.08 | 0.048+/-0.005 |
| *V. komodoensis* | 0.905+/-0.102 | 0.243+/-0.006 | 1.317+/-0.136 | 0.124+/-0.002 | 0.028+/-0.013 |
| *V. melinus* | 3.122+/-0.17 | 0.165+/-0.013 | 2.331+/-0.062 | 1.251+/-0.032 | 0.734+/-0.048 |
| *V. mertensi* | 1.063+/-0.045 | 0.156+/-0.01 | 2.218+/-0.07 | 1.358+/-0.022 | 0.031+/-0.013 |
| *V. mitchelli* | 11.313+/-0.645 | 0.52+/-0.05 | 3.737+/-0.062 | 2.332+/-0.132 | 0.02+/-0.008 |
| *V. panoptes rubidus* | 2.353+/-0.181 | 0.183+/-0.006 | 0.894+/-0.082 | 0.177+/-0.007 | 0.048+/-0.01 |
| *V. prasinus* | 4.604+/-0.407 | 0.454+/-0.03 | 2.72+/-0.225 | 1.509+/-0.067 | 0.071+/-0.005 |
| *V. salvadorii* | 2.353+/-0.136 | 0.054+/-0.006 | 1.305+/-0.058 | 0.559+/-0.015 | 0.117+/-0.018 |
| *V. scalaris* | 4.276+/-0.215 | 0.52+/-0.046 | 3.885+/-0.144 | 2.487+/-0.142 | 0.303+/-0.028 |
| *V. tristis* | 1.821+/-0.17 | 0.021+/-0.002 | 3.081+/-0.082 | 0.898+/-0.022 | 0.043+/-0.013 |
| *V. varius* | 0.815+/-0.102 | 0.118+/-0.007 | 1.465+/-0.089 | 0.711+/-0.027 | 2.549+/-0.056 |
